# MIND diet moderates the associations between cerebrovascular and neurodegenerative disease burden and cognition

**DOI:** 10.3389/fnut.2026.1837406

**Published:** 2026-05-13

**Authors:** Desarae A. Dempsey, Frederick W. Unverzagt, Huiping Xu, Lyndsi Moser, Sujuan Gao, Andrea Avena-Koenigsberger, Evgeny J. Chumin, Karmen K. Yoder, Puja Agarwal, Christy C. Tangney, Daniel O. Clark, Andrew J. Saykin, Shannon L. Risacher

**Affiliations:** 1 Indiana Alzheimer’s Disease Research Center, Indiana University School of Medicine, Indianapolis, IN, United States; 2 Department of Radiology and Imaging Sciences, Indiana University School of Medicine, Indianapolis, IN, United States; 3 Stark Neurosciences Research Institute, Indiana University School of Medicine, Indianapolis, IN, United States; 4 Department of Psychiatry, Indiana University School of Medicine, Indianapolis, IN, United States; 5 Indiana University Center for Aging Research at Regenstrief Institute, Indianapolis, IN, United States; 6 Department of Biostatistics and Health Data Science, Indiana University School of Medicine, Indianapolis, IN, United States; 7 Rush Alzheimer’s Disease Research Center, Rush University Medical Center, Chicago, IL, United States; 8 Department of Internal Medicine, Rush University Medical Center, Chicago, IL, United States; 9 Department of Clinical Nutrition, Rush University Medical Center, Chicago, IL, United States; 10 Department of Family and Preventive Medicine, Rush University Medical Center, Chicago, IL, United States; 11 Division of General Internal Medicine and Geriatrics, Department of Medicine, Indiana University School of Medicine, Indianapolis, IN, United States; 12 Department of Neurology, Indiana University School of Medicine, Indianapolis, IN, United States; 13 Department of Medical and Molecular Genetics, Indiana University School of Medicine, Medical Research and Library Building, Indianapolis, IN, United States; 14 Wake Forest University School of Medicine, Winston-Salem, NC, United States; 15 Wake Forest Alzheimer’s Disease Research Center, Winston-Salem, NC, United States

**Keywords:** Alzheimer’s disease, cerebrovascular disease, cognitive resilience, dementia, Mediterranean-DASH intervention for neurodegenerative delay (MIND) diet, nutrition, plant-based diet

## Abstract

**Introduction:**

The Mediterranean-Dietary Approaches to Stop Hypertension Intervention for Neurodegenerative Delay (MIND) diet is associated with reduced dementia risk, but its role in moderating pathology–cognition relationships in high-risk populations remains unclear. This study examined associations of the MIND diet and Healthy Eating Index (HEI) with cognition and tested whether diet quality modifies the impact of brain pathology on cognitive performance.

**Methods:**

Sixty-six older adults (aged 60–82 years; mean education of 12 years, 65% Black, 73% female) completed MRI and the VioScreen food frequency questionnaire (FFQ). Multivariable linear regression models examined associations between diet scores (MIND, HEI-2020) and cognitive outcomes (cognition composite, memory, executive function), adjusting for age, sex, and education level. Interaction analyses cross-sectionally tested whether diet moderated relationships between structural brain pathology–white matter hyperintensity (WMH), hippocampal, and cortical volumes–and cognition, followed by *post-hoc* simple slopes analyses.

**Results:**

Higher MIND diet scores were independently associated with better memory performance (*p* < 0.05). These were significant interactions between WMH volume and MIND diet score across cognitive outcomes (all p-interactions < 0.05). Greater WMH volume was associated with worse cognitive performance at low (all p < 0.01), but not mean or high MIND diet scores (all *p* > 0.05). Similarly, cortical volume–cognition associations were present in those with low MIND diet scores and attenuated in those with mean or high scores. In contrast, the HEI-2020 score did not modify the effects of brain pathology on cognition, and neither diet quality measure modified hippocampal volume–cognition relationships.

**Conclusion:**

The MIND diet may buffer the cognitive consequences of cerebrovascular pathology and cortical atrophy in older adults at elevated dementia risk and promote cognitive resilience over general healthy eating guidelines.

## 1 Introduction

Alzheimer’s disease (AD) and cerebrovascular disease are the two most common forms of age-associated dementia (1). AD is pathologically characterized by the accumulation of amyloid-β plaques, neurofibrillary tau tangles, and neurodegeneration (2). These pathological changes often begin in the hippocampus and medial temporal structures, leading to memory impairment and progressing to broader cortical involvement and cognitive impairment. Updated diagnostic criteria also recognize vascular and inflammatory processes as important contributors and frequent co-pathologies in AD and related dementias (ADRD) (3). Cerebrovascular disease encompasses stroke and cerebral small vessel disease (cSVD), each of which substantially increases dementia risk. Stroke approximately doubles the risk of dementia (4–6), while cSVD represents the most common vascular contributor to cognitive impairment and dementia (7–9). White matter hyperintensities (WMH), a hallmark of cSVD, are associated with elevated risk of stroke, cognitive impairment (primarily affecting executive function) (9, 10) and ADRD (11, 12). WMH contribute to cognitive decline and dementia through both additive and synergistic effects with AD pathology (11, 12), and the majority of dementias in community-dwelling older adults are due to mixed etiology of AD and vascular pathologies (13, 14).

Structural neuroimaging markers such as WMH and regional brain volumes provide insight into cerebrovascular and neurodegenerative contributions to cognitive impairment and dementia (15). However, measured pathology accounts for only a modest portion of the variability in cognitive decline (16–18). Individuals with comparable levels of pathology can exhibit markedly different cognitive outcomes—a phenomenon referred to as cognitive reserve or resilience (19–22). Cognitive resilience is commonly described as better-than-expected cognitive performance given the degree of underlying brain pathology and reflects the combined influence of genetic factors, life experiences, and environmental exposures that support cognitive function despite neuronal injury. Although intelligence and educational measures have dominated cognitive reserve research, lifestyle factors such as social engagement (23), cognitive activity (24–27), and physical activity (24, 25, 28, 29), are increasingly recognized as contributors to resilience. Nutrition, which may also promote cognitive resilience (30–32), remains comparatively understudied.

Identifying modifiable risk factors that effectively slow cognitive decline independent of brain pathology, particularly among individuals at highest risk for dementia, is of substantial clinical importance. Current treatments, including amyloid-β-targeted therapies, demonstrate only modest efficacy in slowing cognitive decline, and reported clinical trials had limited inclusion of Black (<5%) and underrepresented participants or individuals with low educational attainment (33–36). As a result, the generalizability and effectiveness of these therapies in populations at greatest risk for dementia remains uncertain. In contrast, modifiable lifestyle factors hold considerable promise for dementia prevention and risk reduction (37).

The Mediterranean—Dietary Approaches to Stop Hypertension Intervention for Neurodegenerative Delay (MIND) diet, was developed specifically for dementia prevention (38). Higher adherence to the MIND diet has been associated with slower cognitive decline and reduced incidence of ADRD, often demonstrating stronger associations than other dietary patterns, including the Mediterranean diet (38–41). While healthy dietary patterns such as the MIND diet have been associated with reduced accumulation of certain pathologies, such as reduced WMH (42), stroke (43, 44), hippocampal atrophy (45), hippocampal sclerosis (46), and amyloid-β pathology (47), potentially reflecting “resistance,” less is known about whether the MIND diet supports cognitive performance in the presence of existing pathology. Whether MIND diet strengthens cognitive “resilience” by attenuating the functional consequences of cerebrovascular and neurodegenerative pathology remains largely unexplored.

The present study cross-sectionally examined whether diet quality is associated with cognitive performance and whether diet modifies the relationship between established structural pathology and cognition in older adults at high risk for dementia. Specifically, we tested whether diet quality modified associations of cerebrovascular disease (WMH) and/or neurodegeneration (hippocampal volume, cortical volume) with cognitive function. Dietary scores were assessed using both the MIND diet, designed for neuroprotection, and the Healthy Eating Index-2020 [HEI-2020 (48)], a general measure of diet quality that is not specifically targeted towards brain health. We hypothesized that (1) the MIND diet would be more strongly associated with cognitive performance than the HEI-2020, and (2) a healthier diet would support cognitive resilience and attenuate the negative associations between pathology and cognition.

## 2 Materials and methods

### 2.1 Study population

This study included baseline data from the MINDSpeed study, which was a 2 × 2 randomized factorial design trial designed to evaluate the feasibility of diet modification and cognitive training versus control conditions in older adults with limited formal education (49, 50). Eligible individuals recruited from electronic medical records were English speaking, U.S. born adults, living in Marion County, Indiana, who were ≥ 60 years of age, and reported no more than 12 years of formal education. Exclusion criteria included those with dementia, Alzheimer’s disease, multiple sclerosis, epilepsy, schizophrenia, bipolar disorder, or Parkinson’s disease; stroke or myocardial infarction within the prior 12 months; brain tumor surgery or brain infection; cancer or other illness with short life expectancy according to medical records. Additional details on the population are available in the study design publication (49).

Age, sex, race, and education were self-reported. Education was converted to a continuous variable, with responses of “Some training or classes after high school graduation,” assigned an education of 13 years, to conserve degrees of freedom (df) in linear regression models. Chronic conditions including hypertension, diabetes, stroke, heart attack/myocardial infarction (MI), and Mini-stroke/Transient ischemic attack (TIA) were obtained via electronic medical records. Stroke was based on medical record data unless newly confirmed by clinical radiology (*n* = 1). From the full cohort, “Don’t know” responses (diabetes *n* = 1; stroke *n* = 1; MI *n* = 3; TIA *n* = 8) were recoded as “No” for analyses. Stroke and TIA were combined into one category. Height, weight, and blood pressure were assessed by research assistants. BMI was calculated as [weight in pounds/(height in inches^1^) × 703] based on clinical measurements at baseline. The Geriatric Depression Scale (GDS) (30-point scale) was used to measure depression (51). Higher scores indicate more depressive symptoms. The national and state area deprivation index (ADI) scores were determined from each participant’s current address based on the Neighborhood atlas (52, 53). Higher ADI scores indicate greater disadvantage. To determine Apolipoprotein E (*APOE*) genotype, genomic DNA was extracted from blood samples and restriction enzyme digestion of amplified DNA was performed using the DNeasy Blood and Tissue Kit (Quagen, Inc.) according to the manufacturer’s protocol (54). Individuals were classified as *APOE* ε4 if they carried at least one ε4 allele.

At baseline, the MINDSpeed cohort consisted of 180 participants. Of those, 169 completed the baseline VioScreen FFQ (see below), and 75 completed the baseline MRI scan. Participants who completed an MRI did not significantly differ on demographic, dietary, or cognitive variables investigated, with the exception of significantly higher mean education and a trend toward lower depressive symptoms compared to those who did not complete an MRI. The current study included the overlapping sample of participants who completed the FFQ, MRI scan, and cognitive testing at baseline (*n* = 72). To ensure valid dietary data quality while maintaining an adequate sample size, participants with implausible energy intake were excluded (<600 or >7,000 kcal/day), resulting in a final analytic sample of 66. Sensitivity analyses using more restrictive thresholds [e.g., excluding > 5,000 (*n* = 63) or > 4500 (*n* = 60)] yielded similar results.

### 2.2 Neuroimaging

#### 2.2.1 MRI protocol

MRI was used to measure white matter hyperintensities and brain matter volumes. Scans were acquired on a Siemens 3.0T Prisma (Siemens, Erlangen, Germany) scanner using a 64-channel head coil array with the sequences described previously (49). The T1-weighted Magnetization Prepared Rapid Gradient Echo (MPRAGE) and T2-weighted Fluid-Attenuated Inversion Recovery (FLAIR) sequences were used in the current study.

#### 2.2.2 White matter hyperintensity volume

White matter hyperintensity (WMH) volume was used as a marker of cSVD. Lesions were segmented by the lesion growth algorithm (LGA) from the lesion segmentation toolbox (LST) version 3^2^ for statistical parametric mapping 12 (SPM12), utilizing both the T2-FLAIR and T1-MPRAGE images (55). The LST-LGA algorithm first segments the T1 images into three main tissue classes [cerebrospinal fluid (CSF), grey matter (GM), and white matter (WM)]. This information is then combined with the intensities from the co-registered T2-FLAIR in order to calculate lesion belief maps. By thresholding these maps within a pre-chosen threshold (k), an initial binary lesion map is obtained which is subsequently grown along voxels that appear hyperintense in the T2-FLAIR image. The result is a lesion probability map. The default threshold of 0.5 was used to obtain binary segmentation values from the probability maps, as recommended by the official LST website.

We used a threshold of *k* = 0.25, which has been shown have maximal agreement with total lesion volume (TLV) measured by experienced readers in an older adults with diabetes (56), and provides a more sensitive lesion detection than the pre-set threshold of *k* = 0.3, which has shown maximum agreement in patients with multiple sclerosis (55). Results from the 0.25 threshold and 0.3 threshold showed a correlation of *r* = 1.0. The results were visually checked using the HTML reports, as well as the SPM display tool with overlaid lesion probability maps on the FLAIR and MPRAGE images to determine if voxels were erroneously identified as WMH. One participant was excluded for estimated lesions outside of brain tissue. TLV (mm^3^) was used as the outcome and was highly right skewed. A cube root transformation was applied to normalize the data.

#### 2.2.3 Brain volumes

Hippocampal volume and total cortical volume were used as estimates of brain atrophy. Cortical reconstruction and parcellation using the Desikan-Killiany atlas and volumetric segmentation of subcortical structures was performed in FreeSurfer version 7^2^ using the standard ‘recon-all’ pipeline (57–60). The major standard processing steps include intensity normalization, skull stripping, segmentation of subcortical structures, surface reconstruction, cortical parcellation of white matter and pial surfaces, and registration to a common atlas (61). Both the reconstructed cortical surface parcellations and slice segmentations were visually checked using Freeview. One participant was excluded due to excessive motion, and three participants were excluded for large strokes resulting in distortion.

Total volumes were calculated as the sum of the right and left hemispheres. To account for individual differences in head size, brain volumes were adjusted for total intracranial volume (ICV) using the residual method. Specifically, a linear regression was fit for each volume (y) versus estimated ICV (x), and the residuals used in subsequent analyses. The proportion adjustment method was also evaluated; however, consistent with prior literature, the residual method produced more interpretable and biologically plausible results (62–64).

### 2.3 Dietary information

Information about the participants estimated average diet over the previous 3 months was collected using the validated (65, 66), computerized, semi-quantitative VioScreen food frequency questionnaire (FFQ) from Viocare Technologies . Study personnel prepared the FFQ on an iPad, described the questionnaire and format, and walked them through the first of 20 sections (i.e., Cereals and Breads food group), and then let the subject complete the remainder independently, checking in occasionally to monitor progress and address any questions. The system employs a branching question format, allowing for the quantification of up to 156 food items and allows portion size selection using corresponding images, which is designed to improve recall accuracy and minimize multiple forms of bias (65). The VioScreen automated analysis provides summary data for food groups, nutrients, and dietary pattern scores.

The Healthy Eating Index (HEI) 2020 score, which is based on the current Dietary Guidelines for Americans (DGA) 2020–2025 was an automated output from Viocare technologies (48). The 2020–2025 DGA recommendations fully align with the 2015–2020 guidelines (67). The total score is a sum of 13 components, including 9 adequacy components: total fruits, whole fruits, total vegetables, greens and beans, whole grains, dairy, total protein foods, seafood and plant proteins, and fatty acids, and 4 moderation components: refined grains, sodium, added sugars, and saturated fats. Each component is scored on a scale from 0 to 10 or 0 to 5 based on consumption, and the summed total score ranges from 0 to 100, with higher scores more aligned with the Dietary Guidelines and recommendations. Daily caloric intake (kcal/day), calculated as an automated output of the VioScreen FFQ. Physical activity level was also self-reported via the VioScreen FFQ and categorized as sedentary, low active, active, very active, or extremely active.

The MIND diet score was calculated as described in our previous report (68) based on the direct participant input into the FFQ; the code is publicly available on GitHub: https://github.com/desdemps/VioScreen-MIND-scoring-R. Briefly, the MIND diet score was based on 15 dietary components as previously described (38). The diet includes 10 “brain-healthy” food groups (green leafy vegetables, other vegetables, nuts, berries, beans/legumes, whole grains, fish, poultry, extra virgin olive oil, wine) and five “unhealthy” food groups (red and processed meats, butter and margarine, cheese, pastries and sweets, fried/fast foods) (38). Each of the 15 components was assigned a value of 0, 0.5, or 1, based on pre-defined cut-offs of consumption frequency, with the 5 unhealthy food groups reverse scored so that a 1 corresponds to lower intake. The total MIND diet score was calculated by summing the individual component scores, resulting in a range of scores from 0 to 15 with higher scores indicating greater adherence to the MIND diet pattern.

In this sample, the MIND diet and HEI 2020 scores were positively correlated (Pearson *r* = 0.64; *p* < 0.001). On average, the baseline FFQ and MRI were completed within 2.8 ± 7.6 days (range: 0–41 days).

### 2.4 Cognitive measures

The cognitive tests used in the MINDSpeed study have been described previously (49, 50). All tests were coded such that higher scores indicate better cognitive performance. Raw scores for each test were transformed to standardized z-scores. Composite scores were calculated as the average of the z-scores for tests included. Three composite measures were used: a memory composite, executive composite, and the total cognitive composite.

The memory composite consisted of: 4-trial, 10-item word list learning and delayed recall from the RBANS Update (69); orientation items, and 3-object recall from the MMSE (70). The executive composite score consisted of: 3-letter phonemic fluency (71); 3-category semantic fluency; Symbol Search from the Wechsler Adult Intelligence Scale (WAIS)-IV (72); Trail Making Test Part B, seconds to complete (73); Stroop Color-Word Interference Test, color-word score (74); spell WORLD backwards from the Mini-Mental State Examination (MMSE (70)); 3-step command from the MMSE (70). The overall cognition composite score was then calculated as the average of the memory and executive composite z-scores.

MMSE scores (range 0–30; higher scores indicate better cognition), including both traditional and education-modified scores (75) are reported in the demographics table to facilitate comparison of our sample with other studies, but were not used in analyses.

### 2.5 Statistical analysis

We first examined the relationship between each dietary pattern score (MIND diet score, HEI-2020 score) and cognitive performance using multivariate linear regression models. We tested the relationship with the cognition composite score, followed by domain-specific analyses of memory and executive function composites. All models were adjusted for age (continuous, years), sex (dichotomous: female/male), and education (continuous, years).

For significant associations, additional models were run including neuroimaging measures: WMH, hippocampal, and cortical volumes (all continuous) to test whether the associations were independent of structural brain pathology. Imaging measures were first entered individually and then subsequently included simultaneously within a single model. The variance inflation factor (VIF) for each model was examined to assess multicollinearity. Pearson correlation analyses were used to evaluate associations among the neuroimaging measures. Correlation coefficients were interpreted as follows for association strength: negligible (<0.10), weak (0.10–0.39), moderate (0.40–0.69), strong (0.70–0.89), very strong (0.90–1.00) (76).

We then examined the relationship between structural brain pathology (WMH, hippocampal, and cortical volumes) and cognitive performance (cognition, memory, executive composites). Initial models were fit without interaction terms or main effects of diet scores to characterize main effects. All models adjusted for age, sex, and education level.

Interaction models then tested whether diet modified associations between brain pathology and cognition. Testing interaction effects is the preferred approach to test for potential reserve or resilience factors (19, 22, 77, 78). Separate models were fit for each pathology measure, cognitive outcome, and dietary pattern. Each model included the pathology measure as the independent variable, diet as the moderator, and their interaction term (Pathology x Diet). All interaction models were adjusted for age, sex, and education level.

For significant interaction terms (*p* < 0.05), the adjusted model was probed using *post-hoc* simple slopes analysis to facilitate interpretation (79–81). This approach uses all of the data (not grouped) to estimate the conditional regression slope of the predictor (brain pathology) on the outcome (cognitive performance) at three specific levels of the moderator (diet score): one standard deviation (SD) below the mean (low diet score), the mean diet score, and one SD above the mean (high diet score). The estimates represent the adjusted association between brain pathology and cognition conditional on diet score. The statistics then test whether the slope of the partial regression plot line at that value is significantly different from zero. Both *p*-values and effect sizes were considered in interpretation. The simple slopes were calculated and plotted using the interactions and jtools R packages.

A two tailed *p*-value < 0.05 was considered statistically significant. Throughout the manuscript, β represents the standardized beta estimate, while B represents the unstandardized beta estimate from the model, presented with the 95% confidence intervals (CI). For a coefficient beta, effect sizes between 0.10 and 0.29 are considered small, 0.30–0.49 medium, and 0.50 or greater are large effects (82). Effect size 95% confidence intervals (CI), which were calculated as the beta estimate ± (1.96 × standard error), are considered significant if the interval does not cross zero (83). Error residuals in all linear regression models were examined and passed the assumption of normality. All data were analyzed in R studio (R version 4.4.3).

## 3 Results

### 3.1 Participant demographics

The current study included 66 participants from the MINDSpeed cohort with valid baseline FFQ, MRI, and cognitive testing data. Participant characteristics are summarized in Table 1. The sample was comprised of older adults with mean age of 65.4 ± 4.9 and was predominantly female (73%). A majority of participants identified as Black (65.2%), and half of the sample had a high school education, with 22.7% above and 27.3% below. The cohort exhibited a high burden of cardiometabolic risk factors: 60.0% were obese, 43.9% had diabetes, 80.3% had hypertension, 10.6% had a history of myocardial infarction, and 15.2% had a prior stroke or TIA. The mean MIND diet score was 5.6 ± 1.9 and the mean HEI-2020 score was 59.5 ± 11.4.

**TABLE 1:** Participant demographics.

Participant characteristics, *n* = 66	
Age, years	65.4 ± 4.9 (60.2–82.4)
Sex, Female	72.7%
Education, categorical	
Grade 11 and below	27.3%
Grade 12 or GED	50.0%
Some training or classes after HS grad	22.7%
Education, continuous, years	11.6 ± 1.6 (6–13)
Race	
Black/African American	65.2%
White/Caucasian	30.3%
Other	4.5%
National ADI	79.0 ± 22.4 (13–99)
State ADI	7.2 ± 3.1 (1–10)
*APOE* ε4, positive	43.9%
Cardiovascular components	
Diabetes, yes	43.9%
Hypertension, yes	80.3%
Heart attack/MI, yes	10.6%
Stroke or mini-stroke/TIA, yes	15.2%
Other health variables	
BMI, continuous	33.2 ± 7.9 (20.5–55.4)
BMI, categorical	
Normal weight (<25)	13.8%
Overweight (≥25 to < 30)	26.2%
Obese (≥30)	60.0%
GDS score (30-point scale)	6.1 ± 4.3 (0–20)
Activity level	
Sedentary	25.8%
Low active	39.4%
Active	25.8%
Very active or extremely active	9.1%
Diet scores	
MIND diet score	5.6 ± 1.9 (2.0–11.5)
HEI-2020 score	59.5 ± 11.4 (34.1–88.4)
Energy intake (kcal/day)	2205.6 ± 1188.3 (625.7–6377.5)
Cognitive measures	
Cognition composite	0.04 ± 0.52 (−1.46–1.26)
Memory composite	0.07 ± 0.69 (−1.70–1.42)
Executive composite	−0.02 ± 0.58 (−1.48–1.42)
MMSE, traditional scoring	25.9 ± 2.2 (18–30)
MMSE, modified scoring	26.0 ± 2.2 (18–30)
Neuroimaging variables	
WMH volume, cm^3^†	3.4 ± 7.0 (0–37.1)
Hippocampal volume, mm^3^†	7525.8 ± 735.8 (5903.8–9094.9)
Cortical volume, mm^3^†	351593.2 ± 45143.4 (260338.4–454669.2)
Intracranial volume, mm^3^†	1372773 ± 175285.7 (901880–1833153)

Continuous variables are presented as mean ± standard deviation (range), and categorical variables are presented as percentages. WMH volume reflects total lesion volume prior to cube-root transformation. Hippocampal and cortical volumes represent the sum of left and right hemispheric volumes prior to adjustment for intracranial volume. Cognitive composites were created from multiple cognitive assessments. † Number of excluded participants: WMH volume *n* = 1, Hippocampal, cortical, and intracranial volumes *n* = 4. ADI, area deprivation index; APOE, Apolipoprotein E; BMI, body mass index; COPD, Chronic Obstructive Pulmonary Disorder; GDS, Geriatric Depression Scale; GED, general educational development; HEI, Healthy Eating Index; HS, high school; ICV, intracranial volume; kcal, kilocalories; MI, myocardial infarction; MIND, Mediterranean-Dietary Approaches to Stop Hypertension Intervention for Neurodegenerative Delay; MMSE, Mini-Mental State Examination; TIA, transient ischemic attack; WMH, white matter hyperintensity.

### 3.2 Direct relationship between dietary patterns and cognition

**FIGURE 1:**
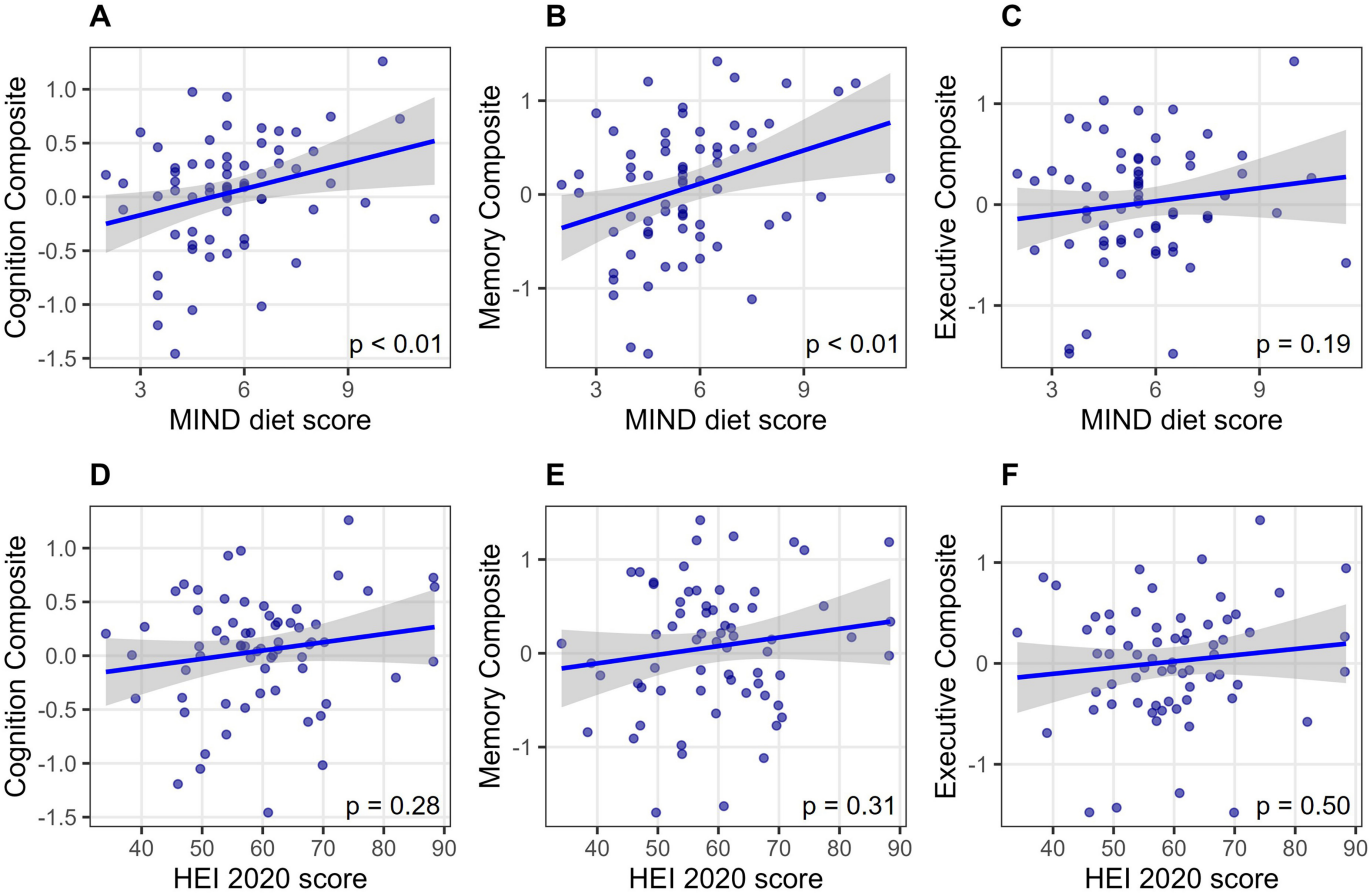
Associations between diet scores and cognitive performance. **(A–C)** Associations between the MIND diet score and cognition composite **(A)**, memory **(B)**, and executive function **(C)**. **(D–F)** The corresponding associations for the HEI-2020 score. Linear regression models were adjusted for age, sex, and education. *P*-values for adjusted associations are displayed in each panel. HEI, Healthy Eating Index; MIND, Mediterranean-Dietary Approaches to Stop Hypertension Intervention for Neurodegenerative Delay.

When adjusted for age, sex, and education, the MIND diet score was positively associated with the overall cognition composite (β = 0.32, 95% CI: 0.09–0.54, *p* = 0.008) (Figure 1A). When analyzed by domain, the MIND diet score was significantly associated with the memory composite (β = 0.34, 95% CI: 0.11–0.58, *p* = 0.005) (Figure 1B), but not the executive function composite (β = 0.16, 95% CI: −0.08 to 0.40, *p* = 0.19) (Figure 1C and Supplementary Table 1). In contrast, the HEI-2020 score was not associated with the cognition composite (β = 0.13, 95% CI: −0.11 to 0.37, *p* = 0.28), memory (β = 0.13, 95% CI: −0.12 to 0.37, *p* = 0.31), or executive function (β = 0.08, 95% CI: −0.16 to 0.33, *p* = 0.50) (Figures 1D–F and Supplementary Table 1).

The association between the MIND diet score and the overall cognition composite remained significant only after adjustment for WMH volume (*p* = 0.02) but was no longer significant when hippocampal volume (*p* = 0.07) or cortical volume (*p* = 0.10) were included (Supplementary Table 2). However, the association between the MIND diet score and the memory composite remained significant after adjustment for each brain pathology measure (WMH, hippocampal, and cortical volumes) entered individually, and simultaneously (all *p* < 0.05) (Supplementary Table 3). All VIFs remained below 1.5, indicating no evidence of problematic multicollinearity. Correlations among neuroimaging measures were weak: WMH was inversely correlated with cortical volume (*r* = −0.32, *p* = 0.01) but not significantly associated with hippocampal volume (*r* = −0.23, *p* = 0.08). Hippocampal and cortical volume were positively correlated (*r* = 0.32, *p* = 0.01).

### 3.3 Direct relationship between structural brain pathology and cognition

Direct associations between each structural pathology measure (WMH, hippocampal, and cortical volumes) and each cognitive composite outcome (cognition, memory, executive) were examined, adjusted for age, sex, and education. Greater WMH volume was associated with worse cognitive performance for the overall cognition composite (β = −0.34, 95% CI: −0.57 to −0.11, *p* = 0.004), and executive function (β = −0.33, 95% CI: −0.57 to −0.10, *p* = 0.006), with a marginal association observed for memory (β = −0.24, 95% CI: −0.49 to 0.01, *p* = 0.056), (Supplementary Table 4). Neither hippocampal volume nor cortical volume were significantly associated with cognitive composite scores (Supplementary Table 4).

### 3.4 Interaction of white matter hyperintensity volume and diet

We then tested whether diet (MIND diet, HEI-2020) modified the relationship between brain pathology and cognition with the full models shown in Supplementary Tables 5–7 and followed up significant interactions with *post-hoc* simple slopes analysis shown in Supplementary Table 8. Each interaction model controlled for age, sex, and education.

**FIGURE 2:**
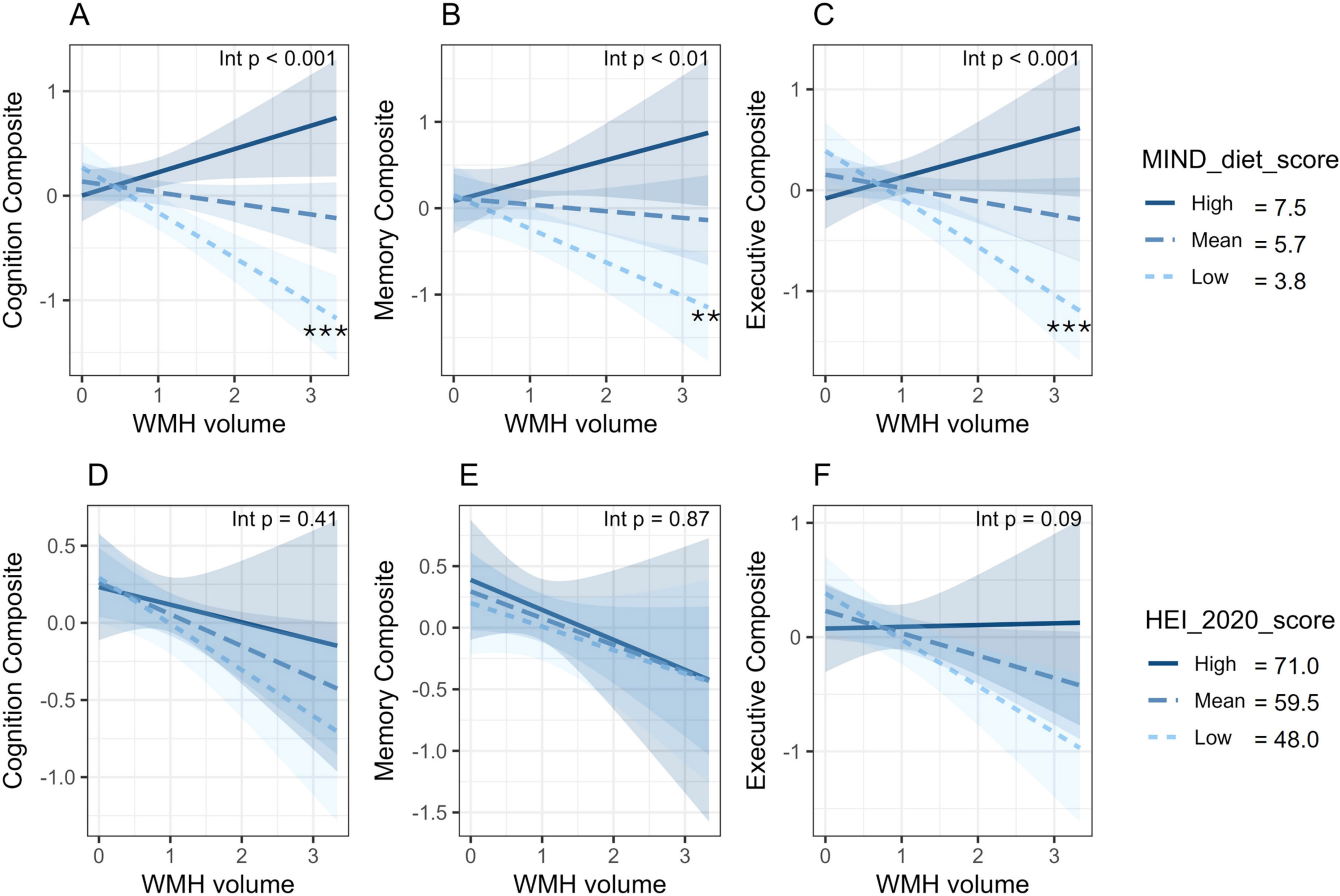
White matter hyperintensity volume x Diet interactions on cognitive performance. **(A–C)** Interactions between WMH volume and MIND diet score for the overall cognition **(A)**, memory **(B)**, and executive function **(C)** composites. **(D–F)** Corresponding interactions for the HEI-2020 score for comparison. *P*-values for overall interaction terms are displayed in the top right corner of each panel. Lines represent estimated associations (simple slopes) between WMH and cognitive performance at low (- 1 SD), mean, and high (+1 SD) levels of diet scores, with shaded areas indicating 95% confidence intervals. Asterisks indicate significant simple slopes (*p < 0.05, **p < 0.01, ***p < 0.001). Models were adjusted for age, sex, and education. Cognitive composite scores were created from multiple cognitive assessments. WMH was cube-root transformed. HEI, Healthy Eating Index; MIND, Mediterranean-Dietary Approaches to Stop Hypertension Intervention for Neurodegenerative Delay; WMH, white matter hyperintensity.

There were significant interactions of the MIND diet score with WMH volume for the overall cognition composite (β = 0.47, 95% CI: 0.28–0.66, *p* < 0.001), memory (β = 0.34, 95% CI: 0.12–0.57, *p* = 0.003) and executive function (β = 0.44, 95% CI: 0.23–0.66, *p* < 0.001) (Figures 2A–C and Table 2). In simple slopes analysis, WMH volume was significantly negatively associated with cognition at the low MIND diet score (−1 SD = 3.8) for the overall cognition composite (β = −0.62, 95% CI: −0.86 to −0.30, *p* < 0.001, Figure 2A), memory (β = −0.43, 95% CI: −0.70 to −0.16, *p* = 0.002, Figure 2B), and executive function (β = −0.62, 95% CI: −0.87 to −0.36, *p* < 0.001, Figure 2C). However, at the mean (5.7) or high (+1 SD = 7.5) MIND diet scores, WMH was not significantly associated with cognitive performance (all *p* > 0.05) (Figures 2A–C). In contrast, the HEI-2020 score did not significantly modify the relationship between WMH volume and cognition for any outcome (all *p* > 0.05) (Figures 2D–F and Table 2). The full interaction models are shown in Supplementary Table 5 and simple slopes in Supplementary Table 8.

**TABLE 2:** Interactions between brain pathology and diet scores predicting cognition.

	Cognition composite		Memory composite		Executive composite	
	β (95% CI)	*p*-value	β (95% CI)	*p*-value	β (95% CI)	*p*-value
MIND diet score						
WMH vol	**−0.15 (−0.35, 0.05)**	**< 0.001**	**−0.08 (−0.32, 0.15)**	**0.001**	**−0.17 (−0.39, 0.05)**	**< 0.001**
Diet score	0.42 (0.22, 0.61)	0.08	0.44 (0.21, 0.67)	0.77	**0.23 (0.01, 0.45)**	**0.01**
WMH vol × Diet score	**0.47 (0.28, 0.66)**	**< 0.001**	**0.34 (0.12, 0.57)**	**0.003**	**0.44 (0.23, 0.66)**	**< 0.001**
Hippocampal vol	0.13 (−0.11, 0.38)	0.66	0.10 (−0.15, 0.35)	0.93	0.11 (−0.14, 0.37)	0.53
Diet score	0.21 (−0.03, 0.45)	0.08	**0.27 (0.03, 0.51)**	**0.03**	0.05 (−0.21, 0.30)	0.70
Hipp vol × Diet score	−0.03 (−0.36, 0.30)	0.86	0.02 (−0.32, 0.36)	0.92	−0.07 (−0.42, 0.28)	0.68
Cortex vol	**0.19 (−0.04, 0.43)**	**0.01**	0.09 (−0.16, 0.34)	0.10	**0.23 (**−**0.02, 0.48)**	**0.02**
Diet score	0.21 (−0.01, 0.44)	0.06	**0.28 (0.03, 0.52)**	**0.02**	0.04 (−0.20, 0.28)	0.69
Cortex vol × Diet score	**−0.30 (−0.56, −0.04)**	**0.02**	−0.22 (−0.48, 0.03)	0.09	−0.27 (−0.54, 0.002)	0.051
HEI-2020 score						
WMH vol	−0.30 (−0.54, −0.05)	0.22	−0.24 (−0.50, 0.03)	0.92	**−0.25 (−0.50, −0.01)**	**0.04**
Diet score	0.13 (−0.11, 0.38)	0.79	0.10 (−0.17, 0.36)	0.56	0.13 (−0.12, 0.37)	0.23
WMH vol × Diet score	0.13 (−0.19, 0.45)	0.41	−0.21 (−0.49, 0.06)	0.13	0.27 (−0.05, 0.59)	0.09
Hippocampal vol	0.16 (−0.10, 0.42)	0.95	0.12 (−0.16, 0.39)	0.92	0.14 (−0.14, 0.41)	0.99
Diet score	0.13 (−0.11, 0.37)	0.27	0.14 (−0.11, 0.39)	0.27	0.06 (−0.18, 0.31)	0.62
Hipp vol × Diet score	0.02 (−0.31, 0.35)	0.91	0.01 (−0.34, 0.35)	0.97	0.03 (−0.31, 0.37)	0.88
Cortex vol	**0.18 (**−**0.07, 0.42)**	**0.04**	0.08 (−0.17, 0.34)	0.07	0.21 (−0.05, 0.46)	0.19
Diet score	0.10 (−0.13, 0.33)	0.38	0.12 (−0.12, 0.37)	0.30	0.02 (−0.22, 0.27)	0.83
Cortex vol × Diet score	−0.23 (−0.47, 0.01)	0.07	−0.22 (−0.48, 0.04)	0.09	−0.13 (−0.39, 0.12)	0.30

Multivariable linear regression models tested whether diet scores (MIND diet, HEI 2020) moderated associations between structural brain pathology (WMH, hippocampal, and cortical volumes), and cognitive performance (overall cognition, memory, and executive composites). Shown are the main effects for diet and brain pathology and their interaction term from models adjusted for age, sex, and education. The values reported are the standardized beta estimate (B), the 95% confidence interval (CI), and *p*-values. Boldface indicates a significant relationship (*p* < 0.05). Cognitive composite scores were created from multiple cognitive assessments. WMH was cube-root transformed. Hippocampal volume and cortical volume were adjusted for intracranial volume (ICV) using the residual method. Full models with covariates are presented in Supplementary Tables 5–7, and *post-hoc* simple slopes for significant interactions are presented in Supplementary Table 8. HEI, Healthy Eating Index; Hipp, Hippocampal; MIND, Mediterranean-Dietary Approaches to Stop Hypertension; vol, volume; WMH, white matter hyperintensity.

### 3.5 Interaction of hippocampal volume and diet

There were no significant interactions between hippocampal volume and either diet score (MIND or HEI-2020) for any cognitive composite outcome (all *p* > 0.05) (Table 2). Full models are presented in Supplementary Table 6.

### 3.6 Interaction of cortical volume and diet

**FIGURE 3:**
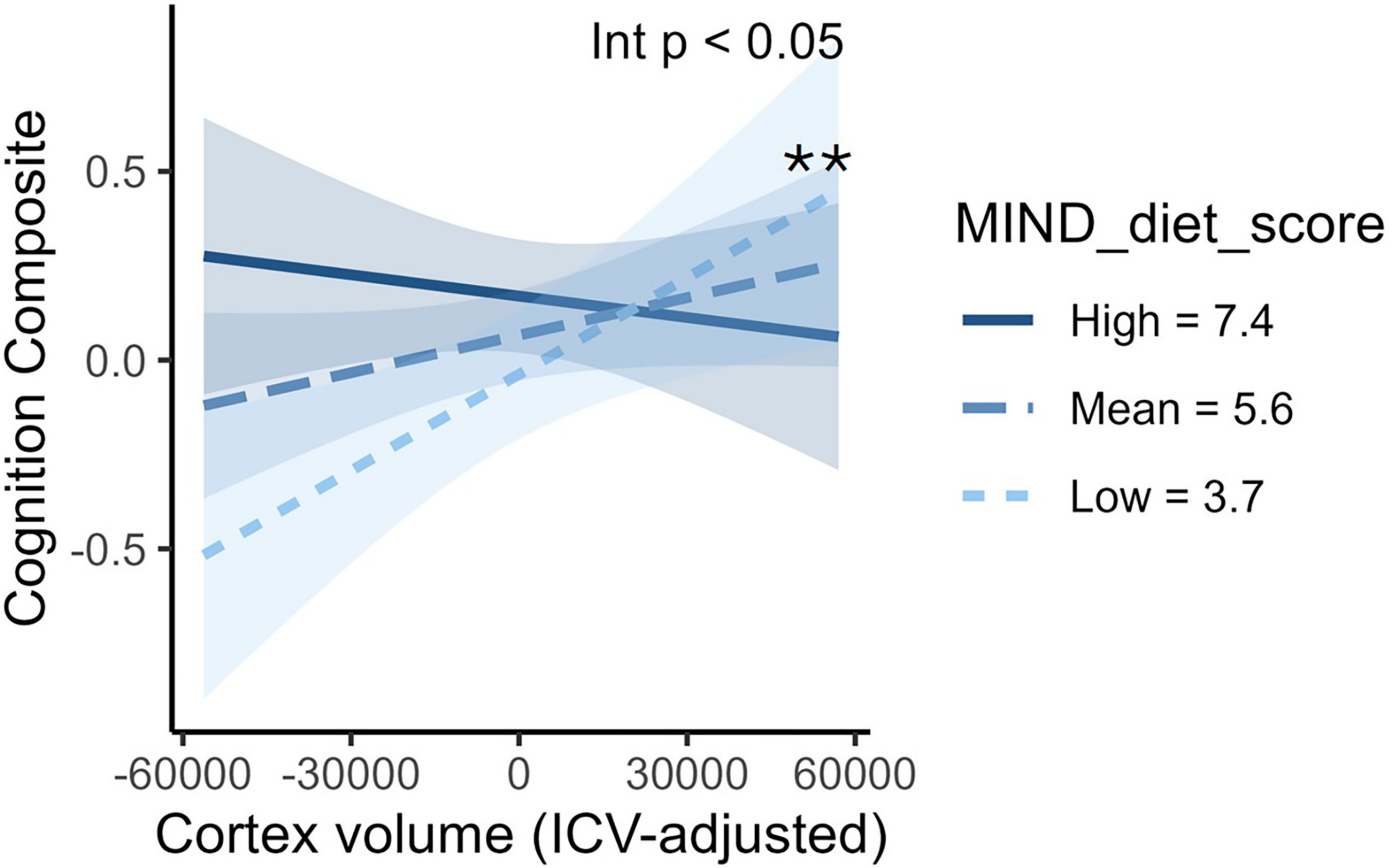
Cortical volume x MIND diet interaction on cognition. The plot shows the interaction between cortical volume and MIND diet score for the overall cognition composite. The *p*-value for the interaction adjusted for age, sex, and education is displayed in the top right corner. Lines represent estimated associations (simple slopes) between cortical volume and cognition at low (- 1 SD), mean, and high (+1 SD) levels of diet scores, with shaded areas indicating 95% confidence intervals. Asterisks indicate significant simple slopes (*p < 0.05, **p < 0.01, ***p < 0.001). The cognition composite was created from multiple cognitive assessments. Cortical volume was adjusted for intracranial volume (ICV) using the residual method. HEI, Healthy Eating Index; ICV, intracranial volume; MIND, Mediterranean-Dietary Approaches to Stop Hypertension Intervention for Neurodegenerative Delay; WMH, white matter hyperintensity.

There was a significant interaction of the MIND diet score with cortical volume for the overall cognition composite (β = −0.30, 95% CI: −0.56 to −0.04, *p* = 0.02), a marginal interaction for executive function (β = −0.27, 95% CI: −0.54 to 0.002, *p* = 0.051), and no significant interaction for memory (β = −0.21, 95% CI: −0.49 to 0.06, *p* = 0.13) (Table 2). In simple slopes analysis, lower cortical volume was significantly associated with worse overall cognition at the low MIND diet score (−1 SD = 3.7) (β = 0.44, 95% CI: 0.12–0.77, *p* = 0.008), but was not significantly associated with cognition at the mean (5.6) or high ( + 1 SD = 7.4) MIND diet scores (*p* > 0.05) (Figure 3). For the HEI-2020 score, there were no significant interactions with cortical volume for any cognition composites (all *p* > 0.05, Table 2). The full interaction models are shown in Supplementary Table 7 and simple slopes in Supplementary Table 8.

## 4 Discussion

In this cross-sectional study of older adults at high risk for dementia, greater adherence to the MIND diet was associated with better memory performance and substantially moderated relationships between structural brain pathology and cognition. The MIND diet showed stronger and more consistent effects than the HEI-2020 dietary pattern. Together, these findings support the concept that adherence to the MIND diet may promote cognitive resilience, enabling individuals to better tolerate the cognitive consequences of structural brain pathology.

### 4.1 Diet and cognition

MIND diet scores were positively associated with better cognitive performance, driven by the memory domain. Each one-point increase in the MIND diet score was associated with an approximately 0.13-point increase in memory performance, when adjusted for age, sex, and education. This association between MIND diet and memory remained independent of structural brain pathology measures, as the association remained significant after adjustment for WMH, hippocampal, and cortical volumes. These findings imply that the MIND diet may support cognitive function, particularly memory, through mechanisms that extend beyond structural brain integrity alone. Rather than merely reducing pathology, dietary quality may enhance compensatory processes or other unexplored mechanisms that allow individuals to maintain cognitive performance despite underlying brain damage.

In contrast, the HEI-2020 score was not significantly associated with cognition. This distinction highlights the specificity of the MIND diet and its components for brain health. While the HEI reflects adherence to general dietary guidelines aimed at cardiometabolic health, the MIND diet emphasizes foods and nutrients with presumed neuroprotective properties. Although the MIND diet and HEI are correlated scores, our findings reinforce the importance of diet composition with specific emphasis on foods known to impact cognitive aging and brain health outcomes.

### 4.2 Pathology and cognition

In models excluding dietary factors, WMH volume was independently associated with worse cognitive performance for the overall cognition composite and executive function, with a marginal association for memory. However, hippocampal and cortical volumes were not independently associated with cognition in adjusted models. These findings suggest that cerebrovascular disease may play a more prominent role in influencing cognitive function in this population, which is characterized by a high vascular and metabolic disease burden.

### 4.3 Diet as a moderator of pathology–cognition relationships

Dietary quality substantially modified the relationship between structural brain pathology and cognition. Higher diet quality attenuated associations between brain pathology–particularly WMH and cortical volume–and cognitive performance, independent of age, sex, and education.

The most consistent and robust moderation effects were observed for the MIND diet and WMH volume. At low MIND diet scores, greater WMH volume was strongly associated with poorer cognitive performance across domains, with medium-to-large effect sizes. However, these negative associations were markedly attenuated and no longer significant at the average and high MIND diet scores. This pattern suggests that individuals with higher adherence to the MIND diet may perform better cognitively than would be expected given their burden of white matter disease. This is clinically relevant in populations with major and minor cerebrovascular disease, such as stroke and cSVD, and in normal aging, as presence of the WMH increases with age even in cognitively normal populations. It also reinforces the clinical relevance of WMH as a potentially modifiable contributor to cognitive decline in vulnerable groups.

A more limited moderation effect was observed for cortical volume. At low MIND diet adherence, greater cortical volume was positively associated with overall cognitive performance with small to moderate effects, whereas this relationship was attenuated at mean and high MIND diet scores. The relationship was observed for overall cognitive performance but was marginally significant for executive function, and not significant for the memory domain. This pattern is consistent with a resilience framework, in which dietary quality may reduce reliance on structural brain integrity for maintaining cognitive function.

In contrast, the HEI-2020 score did not significantly modify WMH volume-cognition or cortical volume-cognition relationships, further highlighting the specificity of the MIND diet for cognitive resilience. This pattern suggests that higher adherence to the MIND diet may more effectively attenuate the cognitive impact of white matter disease and cortical neurodegeneration, compared to general diet quality.

Diet quality did not significantly modify relationships between hippocampal volume and cognition, potentially suggesting that dietary-related resilience may be more effective in buffering the cognitive consequences of vascular and diffuse cortical pathology rather than hippocampal atrophy, a hallmark of AD-related neurodegeneration. Alternatively, the absence of moderation may reflect the underlying pathological profile of this cohort in which hippocampal atrophy may not be driving cognitive function. Thus, its influence on AD-specific processes remains less clear.

Overall, these moderation effects support a cognitive resilience or compensation framework, whereby adherence to the MIND diet enhances the brain’s capacity to compensate for structural damage–particularly cerebrovascular pathology and cortical changes–without proportionate cognitive impairment.

### 4.4 Relevance to previous literature

These findings align with and extend prior observational studies linking the MIND diet to better cognitive outcomes. Longitudinal studies have shown that higher MIND diet adherence is associated with slower cognitive decline (38, 40). While most studies test global cognition, stronger effects have been found for episodic memory, semantic memory, and perceptual speed (38). The present study extends this literature by demonstrating that these benefits persist in a racially diverse, socioeconomically disadvantaged cohort with high risk for dementia, particularly for memory function. Notably, the memory domain specific finding is consistent with results from the 3-month MINDSpeed randomized clinical trial, in which high polyphenol MIND diet snack foods selectively improved memory performance (50). This suggests that memory may be especially sensitive to dietary interventions in populations with elevated risk, giving relevance to the high-antioxidant and polyphenol rich foods emphasized in the MIND diet.

Our results also align with prior work on cognitive resilience. Studies in the Rush Memory and Aging Project (MAP) cohort, which comprises mostly highly educated white participants, found that the MIND diet associated with better cognition independent of post-mortem neuropathology, supportive cognitive resilience (31, 32). While no interaction effects were detected with post-mortem neuropathology (31), differences in study design, pathology measurement (post-mortem vs. *in vivo*), disease burden, and population characteristics likely contribute to this discrepancy. Additional evidence supports dietary resilience against cerebrovascular disease. A recent study found that fish, healthy oils, and nuts attenuated the relationship between diffusion MRI measures of cSVD and cognitive performance, but not for WMH (84). Along with population differences, the results of our study found a significant moderation effect for WMH, potentially suggesting that a comprehensive dietary pattern, rather than these isolated components, may be necessary to buffer the cognitive consequences of substantial white matter disease. This interpretation is supported by studies demonstrating cognitive benefits of the MIND diet in stroke survivors (85, 86) and will be evaluated in an ongoing trial evaluating dietary resilience following stroke (87).

Although we observed no modification of hippocampal volume associations with cognition, findings from the 2-year U.S. Study to Protect Brain Health Through Lifestyle Intervention to Reduce Risk (POINTER) multidomain lifestyle intervention (88) suggest that multidomain lifestyle interventions confer protection against hippocampal vulnerability. Reports indicate that individuals with smaller baseline hippocampal volume or higher tau burden derived greater cognitive benefit from the structured intervention arm (89, 90). While this may be due to effects from the MIND diet, physical activity, and/or cognitive engagement aspects of the trial, these findings support the hypothesis that lifestyle interventions enhance cognitive resilience in those at elevated risk for dementia.

Other studies also suggest that healthy dietary patterns may attenuate the negative effects of age-associated functional white matter connectivity changes on cognition cross-sectionally (91) and longitudinally (92) and may moderate neuropsychiatric symptom relationships to cognition in a sex-specific manner (93). The MIND diet has also been shown to moderate the association between systemic inflammation and both neuroinflammation and cognitive function cross-sectionally (94). Importantly, traditionally studied reserve-related factors, such as education as a proxy, have sometimes (95), but often have not (84, 96, 97), modified the effect of WMH on cognition, while diet has been shown to moderate white matter disease-cognition effects. These traditional reserve variables may act in part through lifestyle factors including diet.

Collectively, this literature supports the role of diet as both an independent contributor to cognitive health and a modifier of pathology-cognition relationships, reinforcing its relevance as a modifiable resilience factor in aging and disease.

### 4.5 Potential biological mechanisms

Several biological mechanisms may underlie the moderation effects observed in this study. The MIND diet emphasizes foods rich in anti-inflammatory and antioxidant compounds, such as vitamins E, C, K, B vitamins, carotenoids, flavonoids and other polyphenols, and healthy fats, that have been linked to better cognitive outcomes (38, 98–100). Damage to the central nervous system and blood vessels results in neuroinflammation, gliosis, and secretion of free radicals (101), which the MIND diet may help neutralize by lowering oxidative stress (102).

WMH reflect chronic microvascular injury, ischemia, inflammation, endothelial dysfunction, and changes in blood-brain barrier (BBB) permeability. Prior work suggests that healthy dietary components may also protect against ischemic damage to the endothelium and BBB (84, 103). Additional plausible mechanisms may include enhanced neurovascular coupling, neuronal membrane fluidity, metabolic efficiency, and synaptic plasticity and transmission, which may help maintain cognitive performance in the face of structural damage. However, these proposed mechanisms remain speculative and were not tested in the present cross-sectional study. Longitudinal, mechanistic studies with more extensive measures are needed to determine whether adherence to the MIND diet is associated with these protective or compensatory processes and how the MIND diet may influence the relationship between pathology and cognitive decline over time.

### 4.6 Strengths and limitations

A strength of this study is the unique MINDSpeed cohort which represents a hard-to-reach, underrepresented population at elevated risk for dementia, including a large proportion of Black females with low educational attainment and socioeconomic status, high vascular risk factors, and overall poorer diet quality (50, 68). Given the comparatively low diet quality in this cohort, the full impact of MIND diet accordance may not be captured. Additional strengths include the use of established neuroimaging biomarkers, domain-specific cognitive outcomes, and the recommended analytical approach for testing reserve variables (22).

Limitations include the modest sample size and cross-sectional design, which preclude causal inference. Additionally, all individuals included in this study were recruited from the Indianapolis metropolitan area, potentially limiting generalizability of the results. Diet and other health factors were self-reported and thus subject to bias. Future studies with larger and more diverse samples, as well as objective measures of dietary intake are warranted. Of note, these structural pathologies are not independent, as WMH has been associated with both cortical and hippocampal volume loss (104, 105). Additionally, the absence of an AD-specific biomarker, such as amyloid-β or phosphorylated tau, limited our ability to disentangle vascular and AD contributions to cognitive function.

### 4.7 Future directions

Future studies should examine dietary resilience across varying types and severities of brain pathology and explore other reserve-related factors. Longitudinal and interventional studies incorporating *in vivo* biomarkers of cerebrovascular disease, AD pathology, and neurodegeneration will be essential to clarify temporal relationships and causal mechanisms. Ongoing and completed intervention studies provide an opportunity to test these hypotheses. While moderation effects were tested using demographics and cardiovascular risk factors in the 3-year MIND diet trial, which showed minimal effects (106, 107), future studies should extend moderation analyses to include biomarkers to better characterize which pathology-cognition relationships are most responsive to dietary intervention. Further, dietary intervention may be most effective in higher risk populations. Secondary analyses of the 3-month MINDSpeed trial outcome data can determine whether individuals with greater baseline cerebrovascular disease derive larger cognitive benefits from high polyphenol snacks or cognitive speed training interventions (50).

## 5 Conclusion

In older adults at high risk for dementia, greater MIND diet accordance was associated with better memory performance and attenuated relationships between structural brain pathology and cognition. Higher adherence to the MIND dietary pattern may more effectively buffer the cognitive consequences of cerebrovascular pathology and cortical neurodegeneration than following general healthy dietary guidelines. These findings highlight MIND dietary intervention as a promising, accessible strategy to support cognitive health and resilience in vulnerable populations.

## Data Availability

The datasets presented in this study are available upon reasonable request to the corresponding author. Requests to access these datasets should be directed to daniclar@iu.edu; asaykin@iu.edu; shannon.risacher@wfusm.edu.

## Footnotes

1
https://surfer.nmr.mgh.harvard.edu/

2
www.applied-statistics.de/lst.html
