# Supplementary material for: MIND diet moderates the associations between cerebrovascular and neurodegenerative disease burden and cognition
Source: Front Nutr. 2026 May 13;13:1837406. doi: 10.3389/fnut.2026.1837406 (PMC13212213; doi:10.3389/fnut.2026.1837406)
Supplement: Supplementary file 1 [file Table_1.docx]

Supplementary Material

# Contents

**Supplemental Table 1.** Associations between diet scores and cognitive composite scores

**Supplemental Table 2.** Associations between MIND diet scores and cognition adjusted for structural brain pathology measures

**Supplemental Table 3.** Associations between MIND diet scores and memory adjusted for structural brain pathology measures

**Supplemental Table 4.** Associations between brain pathology measures and cognition

**Supplemental Table 5.** Interactions between white matter hyperintensity (WMH) volume and diet scores predicting cognition

**Supplemental Table 6.** Interactions between hippocampal volume and diet scores predicting cognition

**Supplemental Table 7.** Interactions between cortex volume and diet scores predicting cognition

**Supplemental Table 8.** Simple slopes analyses for significant pathology x diet score interactions

# Supplementary Tables

| Diet → cognition associations  Models: lm(cognitive composite ~ diet score + covariates) | | | | |
| --- | --- | --- | --- | --- |
| **Outcome** | **Predictor** | **B (95% CI)** | **β (95% CI)** | **p-value** |
|  | **MIND diet score** |  |  |  |
| **Cognition Composite** | (Intercept) | -0.118 (-2.049, 1.814) | -0.024 (-0.286, 0.238) | 0.904 |
|  | MIND diet score | **0.087 (0.024, 0.15)** | **0.315 (0.087, 0.543)** | **0.008** |
|  | Age | -0.024 (-0.048, 0) | -0.226 (-0.453, 0.001) | 0.051 |
|  | Sex (Male) | 0.046 (-0.227, 0.318) | 0.088 (-0.434, 0.61) | 0.737 |
|  | Education | **0.106 (0.031, 0.181)** | **0.317 (0.092, 0.541)** | **0.006** |
| **Memory Composite** | (Intercept) | -1.437 (-4.029, 1.155) | -0.027 (-0.295, 0.24) | 0.272 |
|  | MIND diet score | **0.125 (0.04, 0.21)** | **0.344 (0.111, 0.577)** | **0.005** |
|  | Age | -0.012 (-0.045, 0.02) | -0.089 (-0.321, 0.143) | 0.447 |
|  | Sex (Male) | 0.069 (-0.297, 0.435) | 0.1 (-0.432, 0.633) | 0.707 |
|  | Education | **0.138 (0.037, 0.239)** | **0.314 (0.084, 0.543)** | **0.008** |
| **Executive Composite** | (Intercept) | 1.202 (-1.058, 3.463) | -0.011 (-0.288, 0.266) | 0.292 |
|  | MIND diet score | 0.049 (-0.024, 0.123) | 0.161 (-0.08, 0.402) | 0.186 |
|  | Age | **-0.036 (-0.064, -0.007)** | **-0.303 (-0.543, -0.063)** | **0.014** |
|  | Sex (Male) | 0.023 (-0.296, 0.342) | 0.04 (-0.511, 0.591) | 0.886 |
|  | Education | 0.074 (-0.014, 0.162) | 0.2 (-0.037, 0.437) | 0.097 |
|  | | | | |
|  | **HEI 2020 score** |  |  |  |
| **Cognition Composite** | (Intercept) | 0.025 (-2.12, 2.169) | 0.008 (-0.267, 0.282) | 0.982 |
|  | HEI 2020 score | 0.006 (-0.005, 0.017) | 0.129 (-0.109, 0.367) | 0.283 |
|  | Age | -0.023 (-0.048, 0.003) | -0.214 (-0.454, 0.026) | 0.079 |
|  | Sex (Male) | -0.014 (-0.297, 0.268) | -0.028 (-0.569, 0.513) | 0.919 |
|  | **Education** | **0.1 (0.021, 0.179)** | **0.299 (0.063, 0.535)** | **0.014** |
| **Memory Composite** | (Intercept) | -1.168 (-4.071, 1.734) | 0.008 (-0.274, 0.291) | 0.424 |
|  | HEI 2020 score | 0.008 (-0.007, 0.022) | 0.126 (-0.119, 0.371) | 0.307 |
|  | Age | -0.011 (-0.045, 0.024) | -0.077 (-0.325, 0.17) | 0.534 |
|  | Sex (Male) | -0.021 (-0.404, 0.361) | -0.031 (-0.588, 0.526) | 0.912 |
|  | **Education** | **0.13 (0.023, 0.236)** | **0.295 (0.052, 0.538)** | **0.018** |
| **Executive Composite** | (Intercept) | 1.217 (-1.197, 3.632) | 0.004 (-0.275, 0.282) | 0.317 |
|  | HEI 2020 score | 0.004 (-0.008, 0.016) | 0.083 (-0.159, 0.325) | 0.495 |
|  | Age | **-0.035 (-0.063, -0.006)** | **-0.295 (-0.539, -0.051)** | **0.019** |
|  | Sex (Male) | -0.008 (-0.326, 0.311) | -0.013 (-0.563, 0.537) | 0.962 |
|  | Education | 0.071 (-0.018, 0.159) | 0.19 (-0.049, 0.43) | 0.117 |

**Supplemental Table 1.** Associations between diet scores and cognitive composite scores

Multivariable linear regression models examined associations between diet scores (MIND and HEI-2020) and cognitive composite outcomes (overall cognition, memory, executive function). All models were adjusted for age, sex, and education. Values represent the unstandardized (B) and standardized (β) beta coefficients with 95% confidence intervals (CI) and p-values for each predictor in each model. Boldface indicates significant predictor (p<0.05).

Higher MIND diet scores significantly associated with the cognition and memory composites, but not the executive function composite. HEI 2020 scores were not significantly associated with any cognitive composite scores.

| MIND diet → overall cognition associations covariated for brain pathology  Models: lm(cognition composite ~ MIND diet score + covariates + brain pathology measure(s)) | | | | |
| --- | --- | --- | --- | --- |
| **Outcome** | **Predictor** | **B (95% CI)** | **β (95% CI)** | **p-value** |
| **Cognition Composite** | (Intercept) | 0.177 (-1.712, 2.065) | -0.051 (-0.304, 0.203) | 0.852 |
|  | MIND diet score | **0.078 (0.016, 0.141)** | **0.277 (0.055, 0.499)** | **0.015** |
|  | Age | -0.021 (-0.044, 0.002) | -0.198 (-0.417, 0.02) | 0.075 |
|  | Sex (Male) | 0.096 (-0.169, 0.362) | 0.183 (-0.322, 0.689) | 0.471 |
|  | Education | **0.087 (0.013, 0.16)** | **0.259 (0.039, 0.479)** | **0.022** |
|  | WMH volume | **-0.212 (-0.368, -0.057)** | **-0.304 (-0.527, -0.081)** | **0.008** |
| **Cognition Composite** | (Intercept) | 0.407 (-1.361, 2.174) | 0.028 (-0.237, 0.293) | 0.647 |
|  | MIND diet score | 0.054 (-0.005, 0.114) | 0.212 (-0.021, 0.446) | 0.074 |
|  | Age | **-0.026 (-0.048, -0.005)** | **-0.282 (-0.514, -0.051)** | **0.018** |
|  | Sex (Male) | -0.051 (-0.306, 0.204) | -0.108 (-0.649, 0.432) | 0.689 |
|  | Education | **0.093 (0.02, 0.165)** | **0.31 (0.068, 0.551)** | **0.013** |
|  | Hippo volume | 0.0001 (-0.0001, 0.0003) | 0.135 (-0.106, 0.375) | 0.266 |
| **Cognition Composite** | (Intercept) | 0.092 (-1.678, 1.861) | 0.05 (-0.215, 0.315) | 0.918 |
|  | MIND diet score | 0.05 (-0.01, 0.109) | 0.195 (-0.039, 0.428) | 0.101 |
|  | Age | **-0.023 (-0.045, -0.001)** | **-0.246 (-0.484, -0.008)** | **0.043** |
|  | Sex (Male) | -0.092 (-0.352, 0.169) | -0.194 (-0.746, 0.358) | 0.484 |
|  | Education | **0.104 (0.036, 0.172)** | **0.347 (0.119, 0.574)** | **0.003** |
|  | Cortex volume | 2.9x10^-6^ (-1.3x10^-6^, -7.1x10^-6^) | 0.169 (-0.073, 0.411) | 0.167 |
| **Cognition Composite** | (Intercept) | 0.45 (-1.364, 2.265) | 0.018 (-0.25, 0.285) | 0.621 |
|  | MIND diet score | 0.045 (-0.016, 0.107) | 0.173 (-0.063, 0.409) | 0.147 |
|  | Age | **-0.022 (-0.045, 0)** | **-0.238 (-0.474, -0.001)** | **0.049** |
|  | Sex (Male) | -0.032 (-0.302, 0.238) | -0.067 (-0.634, 0.5) | 0.814 |
|  | Education | **0.083 (0.01, 0.156)** | **0.277 (0.034, 0.52)** | **0.026** |
|  | WMH volume | -0.153 (-0.318, 0.011) | -0.235 (-0.487, 0.017) | 0.067 |
|  | Hippo volume | 0.0001 (-0.0001, 0.0003) | 0.074 (-0.179, 0.327) | 0.561 |
|  | Cortex volume | 1.1x10^-6^ (-3.6x10^-6^, 5.8x10^-6^) | 0.063 (-0.207, 0.333) | 0.642 |

**Supplemental Table 2.** Associations between MIND diet scores and cognition adjusted for structural brain pathology measures

Multivariable linear regression models examined associations between the MIND diet score and the overall cognitive composite, adjusted for age, sex, education, and each brain pathology measure (white matter hyperintensity [WMH] volume, hippocampal volume, cortical volume), individiually, and then together. Values represent the unstandardized (B) and standardized (β) beta coefficients with 95% confidence intervals (CI) and p-values for each predictor in each model. Boldface indicates significant predictor (p<0.05).

Cognitive composite scores were created from multiple cognitive assessments. WMH was cube-root transformed. Hippocampal volume and cortical volume were adjusted for intracranial volume using the residual method.

MIND diet score remained a significant predictor of overall cognition when WMH was added to the model, and became marginally significant when hippocampal volume or cortical volume were added, and non-significant when all three were added together.

| MIND diet → memory associations covariated for brain pathology  Models: lm(memory composite ~ MIND score + covariates + brain pathology measure(s)) | | | | |
| --- | --- | --- | --- | --- |
| **Outcome** | **Predictor** | **B (95% CI)** | **β (95% CI)** | **p-value** |
| **Memory Composite** | (Intercept) | -1.349 (-3.957, 1.258) | -0.054 (-0.32, 0.213) | 0.305 |
|  | MIND diet score | **0.126 (0.039, 0.213)** | **0.339 (0.105, 0.572)** | **0.005** |
|  | Age | -0.009 (-0.041, 0.023) | -0.064 (-0.294, 0.166) | 0.581 |
|  | Sex (Male) | 0.134 (-0.233, 0.501) | 0.194 (-0.337, 0.725) | 0.468 |
|  | Education | **0.125 (0.023, 0.226)** | **0.283 (0.052, 0.514)** | **0.017** |
|  | WMH volume | -0.18 (-0.395, 0.036) | -0.195 (-0.43, 0.039) | 0.100 |
| **Memory Composite** | (Intercept) | -1.088 (-3.593, 1.416) | 0.033 (-0.24, 0.306) | 0.388 |
|  | MIND diet score | **0.094 (0.01, 0.179)** | **0.27 (0.028, 0.511)** | **0.029** |
|  | Age | -0.014 (-0.045, 0.017) | -0.11 (-0.349, 0.129) | 0.359 |
|  | Sex (Male) | -0.082 (-0.444, 0.279) | -0.127 (-0.685, 0.431) | 0.650 |
|  | Education | **0.136 (0.033, 0.239)** | **0.329 (0.08, 0.579)** | **0.011** |
|  | Hippo volume | 0.0001 (-0.0002, 0.0004) | 0.097 (-0.151, 0.345) | 0.439 |
| **Memory Composite** | (Intercept) | -1.322 (-3.85, 1.205) | 0.042 (-0.233, 0.318) | 0.299 |
|  | MIND diet score | **0.092 (0.006, 0.177)** | **0.262 (0.018, 0.505)** | **0.035** |
|  | Age | -0.012 (-0.044, 0.02) | -0.096 (-0.343, 0.152) | 0.441 |
|  | Sex (Male) | -0.107 (-0.479, 0.266) | -0.165 (-0.739, 0.409) | 0.568 |
|  | Education | **0.147 (0.05, 0.245)** | **0.358 (0.121, 0.594)** | **0.004** |
|  | Cortex volume | 1.8x10^-6^ (-4.2x10^-6^, 7.8x10^-6^) | 0.075 (-0.177, 0.327) | 0.553 |
| **Memory Composite** | (Intercept) | -1.116 (-3.749, 1.517) | 0.01 (-0.273, 0.292) | 0.399 |
|  | MIND diet score | **0.099 (0.009, 0.188)** | **0.275 (0.026, 0.524)** | **0.031** |
|  | Age | -0.011 (-0.043, 0.021) | -0.087 (-0.337, 0.163) | 0.489 |
|  | Sex (Male) | -0.024 (-0.416, 0.368) | -0.036 (-0.636, 0.563) | 0.903 |
|  | Education | **0.128 (0.022, 0.234)** | **0.311 (0.054, 0.568)** | **0.019** |
|  | WMH volume | -0.126 (-0.365, 0.112) | -0.141 (-0.407, 0.125) | 0.293 |
|  | Hippo volume | 0.0001 (-0.0002, 0.0004) | 0.086 (-0.181, 0.354) | 0.519 |
|  | Cortex volume | -3.5x10^-7^ (-7.2x10^-6^, 6.5x10^-6^) | -0.015 (-0.3, 0.271) | 0.919 |

**Supplemental Table 3.** Associations between MIND diet scores and memory adjusted for structural brain pathology measures

Multivariable linear regression models examined associations between the MIND diet score and the memory composite, adjusted for age, sex, education, and each brain pathology measure (white matter hyperintensity [WMH] volume, hippocampal volume, cortical volume), individiually, and then together. Values represent the unstandardized (B) and standardized (β) beta coefficients with 95% confidence intervals (CI) and p-values for each predictor in each model. Boldface indicates significant predictor (p<0.05).

Cognitive composite scores were created from multiple cognitive assessments. WMH was cube-root transformed. Hippocampal volume and cortical volume were adjusted for intracranial volume using the residual method.

MIND diet score remained a significant predictor of memory when WMH, hippocampal, or cortical volume were added to the model, individually or together.

| Pathology → cognition associations  Models: lm(cognition composite ~ brain pathology + covariates) | | | | |
| --- | --- | --- | --- | --- |
| **Outcome** | **Predictor** | **B (95% CI)** | **β (95% CI)** | **p-value** |
|  | **White matter hyperintensity (WMH) volume** | | |  |
| **Cognition Composite** | (Intercept) | 0.79 (-1.11, 2.69) | -0.01 (-0.272, 0.252) | 0.409 |
|  | WMH volume | **-0.238 (-0.399, -0.077)** | **-0.34 (-0.571, -0.11)** | **0.004** |
|  | Age | -0.021 (-0.046, 0.003) | -0.203 (-0.431, 0.025) | 0.080 |
|  | Sex (Male) | 0.019 (-0.25, 0.289) | 0.036 (-0.476, 0.549) | 0.888 |
|  | Education | **0.079 (0.003, 0.156)** | **0.236 (0.008, 0.465)** | **0.043** |
| **Memory Composite** | (Intercept) | -0.363 (-3.031, 2.305) | -0.004 (-0.284, 0.276) | 0.786 |
|  | WMH volume | -0.22 (-0.447, 0.006) | -0.24 (-0.486, 0.006) | 0.056 |
|  | Age | -0.01 (-0.044, 0.024) | -0.069 (-0.313, 0.174) | 0.572 |
|  | Sex (Male) | 0.01 (-0.369, 0.388) | 0.014 (-0.533, 0.562) | 0.959 |
|  | Education | **0.112 (0.005, 0.22)** | **0.255 (0.011, 0.499)** | **0.041** |
| **Executive Composite** | (Intercept) | 1.944 (-0.181, 4.069) | -0.014 (-0.279, 0.252) | 0.072 |
|  | WMH volume | **-0.255 (-0.436, -0.075)** | **-0.331 (-0.565, -0.097)** | **0.006** |
|  | Age | **-0.033 (-0.06, -0.006)** | **-0.285 (-0.516, -0.054)** | **0.016** |
|  | Sex (Male) | 0.028 (-0.273, 0.33) | 0.049 (-0.47, 0.569) | 0.851 |
|  | Education | 0.046 (-0.04, 0.132) | 0.124 (-0.107, 0.356) | 0.287 |
|  | | | | |
|  | **Hippocampal volume** | |  |  |
| **Cognition Composite** | (Intercept) | 0.765 (-0.992, 2.522) | 0.058 (-0.209, 0.326) | 0.387 |
|  | Hippo volume | 0.0001 (-0.0001, 0.0003) | 0.134 (-0.111, 0.38) | 0.277 |
|  | Age | **-0.027 (-0.049, -0.005)** | **-0.285 (-0.521, -0.048)** | **0.019** |
|  | Sex (Male) | -0.107 (-0.359, 0.146) | -0.226 (-0.761, 0.309) | 0.402 |
|  | Education | **0.091 (0.017, 0.164)** | **0.302 (0.055, 0.548)** | **0.017** |
| **Memory Composite** | (Intercept) | -0.463 (-2.988, 2.061) | 0.071 (-0.209, 0.351) | 0.715 |
|  | Hippo volume | 0.0001 (-0.0002, 0.0004) | 0.096 (-0.16, 0.353) | 0.455 |
|  | Age | -0.015 (-0.046, 0.017) | -0.113 (-0.361, 0.134) | 0.363 |
|  | Sex (Male) | -0.179 (-0.542, 0.184) | -0.276 (-0.836, 0.284) | 0.328 |
|  | Education | **0.132 (0.025, 0.238)** | **0.319 (0.062, 0.577)** | **0.016** |
| **Executive Composite** | (Intercept) | 1.994 (-0.071, 4.059) | 0.016 (-0.26, 0.293) | 0.058 |
|  | Hippo volume | 0.0001 (-0.0001, 0.0003) | 0.12 (-0.133, 0.373) | 0.346 |
|  | Age | **-0.039 (-0.065, -0.013)** | **-0.363 (-0.607, -0.119)** | **0.004** |
|  | Sex (Male) | -0.034 (-0.331, 0.263) | -0.064 (-0.616, 0.489) | 0.818 |
|  | Education | 0.049 (-0.037, 0.136) | 0.145 (-0.109, 0.399) | 0.259 |
|  | | | | |
|  | **Cortex volume** | |  |  |
| **Cognition Composite** | (Intercept) | 0.391 (-1.368, 2.149) | 0.08 (-0.186, 0.347) | 0.658 |
|  | Cortex volume | 3.3x10^-6^ (-9.3x10^-7^, 7.6x10^-6^) | 0.191 (-0.053, 0.435) | 0.123 |
|  | **Age** | **-0.023 (-0.045, -0.0001)** | **-0.243 (-0.484, -0.001)** | **0.049** |
|  | Sex (Male) | -0.147 (-0.403, 0.108) | -0.312 (-0.853, 0.229) | 0.254 |
|  | **Education** | **0.102 (0.032, 0.171)** | **0.339 (0.108, 0.569)** | **0.005** |
| **Memory Composite** | (Intercept) | -0.771 (-3.323, 1.781) | 0.083 (-0.198, 0.365) | 0.548 |
|  | Cortex volume | 2.5x10^-6^ (-3.7x10^-6^, 8.6x10^-6^) | 0.104 (-0.154, 0.363) | 0.422 |
|  | Age | -0.012 (-0.045, 0.021) | -0.091 (-0.346, 0.164) | 0.476 |
|  | Sex (Male) | -0.209 (-0.58, 0.162) | -0.323 (-0.895, 0.249) | 0.263 |
|  | Education | **0.143 (0.043, 0.243)** | **0.347 (0.103, 0.591)** | **0.006** |
| **Executive Composite** | (Intercept) | 1.552 (-0.503, 3.607) | 0.041 (-0.233, 0.314) | 0.136 |
|  | Cortex volume | 4.1x10^-6^ (-8.1x10^-7^, 9.1x10^-6^) | 0.21 (-0.041, 0.46) | 0.100 |
|  | **Age** | **-0.034 (-0.06, -0.007)** | **-0.316 (-0.564, -0.068)** | **0.013** |
|  | Sex (Male) | -0.085 (-0.384, 0.214) | -0.158 (-0.714, 0.397) | 0.570 |
|  | Education | 0.06 (-0.021, 0.141) | 0.176 (-0.06, 0.413) | 0.141 |

**Supplemental Table 4.** Associations between brain pathology measures and cognition

Multivariable linear regression models examined associations brain pathology measures (white matter hyperintensity [WMH] volume, hippocampal volume, cortical volume) and cognitive composites (cognition, memory, executive) outcomes adjusting for age, sex, and education. Values represent the unstandardized (B) and standardized (β) beta coefficients with 95% confidence intervals (CI) and p-values for each predictor in each model. Boldface indicates significant predictor (p<0.05).

Cognitive composite scores were created from multiple cognitive assessments. WMH was cube-root transformed. Hippocampal volume and cortical volume were adjusted for intracranial volume using the residual method.

WMH volume was a significant predictor of overall cognition, and executive function composite scores, and marginally associated with memory. Hippocampal and cortical volumes were not significant predictors of cognition.

| White matter hyperintensity (WMH) volume x diet score → cognition interactions  Models: lm(cognitive composite ~ WMH volume x diet score + covariates) | | | | |
| --- | --- | --- | --- | --- |
| **Outcome** | **Predictor** | **B (95% CI)** | **β (95% CI)** | **p-value** |
|  | **WMH x MIND diet score interactions** | | |  |
| **Cognition Composite** | (Intercept) | 0.722 (-0.902, 2.346) | -0.046 (-0.261, 0.17) | 0.377 |
|  | WMH volume | **-1.107 (-1.5, -0.714)** | **-0.15 (-0.35, 0.05)** | **<0.001** |
|  | MIND diet score | -0.073 (-0.155, 0.01) | 0.416 (0.219, 0.614) | 0.083 |
|  | Age | **-0.021 (-0.041, -0.002)** | **-0.202 (-0.388, -0.015)** | **0.034** |
|  | Sex (Male) | 0.221 (-0.011, 0.454) | 0.421 (-0.021, 0.863) | 0.061 |
|  | Education | **0.106 (0.043, 0.169)** | **0.317 (0.128, 0.506)** | **0.001** |
|  | WMH volume x MIND | **0.176 (0.103, 0.249)** | **0.469 (0.275, 0.662)** | **<0.001** |
| **Memory Composite** | (Intercept) | -0.827 (-3.294, 1.639) | -0.05 (-0.299, 0.2) | 0.505 |
|  | WMH volume | **-1.035 (-1.632, -0.437)** | **-0.084 (-0.315, 0.148)** | **0.001** |
|  | MIND diet score | -0.018 (-0.143, 0.107) | 0.44 (0.211, 0.668) | 0.770 |
|  | Age | -0.009 (-0.039, 0.021) | -0.066 (-0.282, 0.149) | 0.540 |
|  | Sex (Male) | 0.253 (-0.099, 0.606) | 0.367 (-0.144, 0.877) | 0.156 |
|  | Education | **0.143 (0.047, 0.239)** | **0.325 (0.107, 0.543)** | **0.004** |
|  | WMH volume x MIND | **0.168 (0.058, 0.279)** | **0.341 (0.117, 0.565)** | **0.003** |
| **Executive Composite** | (Intercept) | 2.272 (0.298, 4.246) | -0.023 (-0.261, 0.215) | 0.025 |
|  | WMH volume | **-1.179 (-1.657, -0.701)** | **-0.173 (-0.393, 0.048)** | **<0.001** |
|  | MIND diet score | **-0.127 (-0.227, -0.027)** | **0.231 (0.013, 0.448)** | **0.014** |
|  | Age | **-0.034 (-0.058, -0.01)** | **-0.287 (-0.492, -0.081)** | **0.007** |
|  | Sex (Male) | 0.189 (-0.093, 0.472) | 0.326 (-0.16, 0.813) | 0.185 |
|  | Education | 0.069 (-0.008, 0.146) | 0.187 (-0.021, 0.395) | 0.077 |
|  | WMH volume x MIND | **0.184 (0.095, 0.273)** | **0.443 (0.23, 0.657)** | **<0.001** |
|  | | | | |
|  | **WMH x HEI 2020 score interactions** | | |  |
| **Cognition Composite** | (Intercept) | 0.762 (-1.45, 2.973) | -0.015 (-0.279, 0.25) | 0.493 |
|  | WMH volume | -0.685 (-1.801, 0.431) | -0.295 (-0.543, -0.047) | 0.224 |
|  | HEI 2020 score | -0.003 (-0.023, 0.017) | 0.132 (-0.114, 0.378) | 0.793 |
|  | Age | -0.02 (-0.045, 0.004) | -0.191 (-0.421, 0.04) | 0.104 |
|  | Sex (Male) | 0.063 (-0.219, 0.344) | 0.119 (-0.417, 0.655) | 0.658 |
|  | Education | **0.085 (0.006, 0.163)** | **0.253 (0.019, 0.487)** | **0.035** |
|  | WMH volume x HEI | 0.008 (-0.012, 0.028) | 0.132 (-0.189, 0.453) | 0.414 |
| **Memory Composite** | (Intercept) | -0.934 (-4.056, 2.187) | -0.014 (-0.298, 0.27) | 0.551 |
|  | WMH volume | -0.083 (-1.659, 1.493) | -0.237 (-0.503, 0.03) | 0.917 |
|  | HEI 2020 score | 0.008 (-0.02, 0.036) | 0.095 (-0.168, 0.359) | 0.564 |
|  | Age | -0.008 (-0.043, 0.026) | -0.058 (-0.306, 0.19) | 0.640 |
|  | Sex (Male) | 0.026 (-0.372, 0.424) | 0.038 (-0.538, 0.613) | 0.896 |
|  | Education | 0.11 (-0.001, 0.221) | 0.25 (-0.001, 0.501) | 0.051 |
|  | WMH volume x HEI | -0.002 (-0.03, 0.025) | -0.028 (-0.373, 0.316) | 0.870 |
| **Executive Composite** | (Intercept) | **2.457 (0.02, 4.895)** | **-0.01 (-0.274, 0.255)** | **0.048** |
|  | WMH volume | **-1.287 (-2.518, -0.057)** | **-0.253 (-0.501, -0.005)** | **0.041** |
|  | HEI 2020 score | -0.013 (-0.035, 0.009) | 0.126 (-0.12, 0.371) | 0.226 |
|  | Age | **-0.032 (-0.059, -0.005)** | **-0.276 (-0.507, -0.046)** | **0.020** |
|  | Sex (Male) | 0.099 (-0.211, 0.41) | 0.171 (-0.364, 0.706) | 0.525 |
|  | Education | 0.06 (-0.027, 0.146) | 0.161 (-0.073, 0.395) | 0.173 |
|  | WMH volume x HEI | 0.018 (-0.003, 0.04) | 0.273 (-0.048, 0.594) | 0.094 |

**Supplemental Table 5.** Interactions between white matter hyperintensity (WMH) volume and diet scores predicting cognition

Multivariable linear regression models tested interaction terms between WMH volume and diet scores (MIND, HEI-2020) predicting cognitive composite outcomes (cognition, memory, executive function), adjusted for age, sex, and education. Values represent the unstandardized (B) and standardized (β) beta coefficients with 95% confidence intervals (CI) and p-values for each predictor in each model. Boldface indicates significant predictor (p<0.05).

Cognitive composite scores were created from multiple cognitive assessments. WMH was cube-root transformed. Hippocampal volume and cortical volume were adjusted for intracranial volume using the residual method.

WMH volume significantly interacted with the MIND diet score in predicting the overall cognition, memory, and executive function composites. WMH volume only significantly interacted with the HEI 2020 score in predicting executive function, but but memory or overall cognition.

| Hippocampal volume x diet score → cognition interactions  Models: lm(cognitive composite ~ hippocampal volume x diet score + covariates) | | | | |
| --- | --- | --- | --- | --- |
| **Outcome** | **Predictor** | **B (95% CI)** | **β (95% CI)** | **p-value** |
|  | **Hippocampal volume x MIND diet score interactions** | | |  |
| **Cognition Composite** | (Intercept) | 0.426 (-1.371, 2.224) | 0.025 (-0.244, 0.294) | 0.636 |
|  | Hippo volume | 0.0002 (-0.001, 0.001) | 0.132 (-0.112, 0.376) | 0.657 |
|  | MIND diet score | 0.054 (-0.006, 0.114) | 0.211 (-0.025, 0.447) | 0.076 |
|  | Age | **-0.027 (-0.049, -0.005)** | **-0.285 (-0.522, -0.049)** | **0.019** |
|  | Sex (Male) | -0.046 (-0.31, 0.218) | -0.098 (-0.656, 0.461) | 0.727 |
|  | Education | **0.093 (0.02, 0.166)** | **0.309 (0.065, 0.553)** | **0.014** |
|  | Hipp vol x MIND | 0.00001 (-0.0002, 0.0001) | -0.029 (-0.357, 0.299) | 0.858 |
| **Memory Composite** | (Intercept) | -1.104 (-3.652, 1.443) | 0.035 (-0.243, 0.312) | 0.389 |
|  | Hippo volume | 0.0001 (-0.001, 0.001) | 0.098 (-0.154, 0.35) | 0.929 |
|  | MIND diet score | **0.094 (0.009, 0.18)** | **0.27 (0.026, 0.514)** | **0.031** |
|  | Age | -0.014 (-0.045, 0.017) | -0.109 (-0.353, 0.136) | 0.377 |
|  | Sex (Male) | -0.087 (-0.46, 0.287) | -0.133 (-0.709, 0.443) | 0.644 |
|  | Education | **0.136 (0.032, 0.24)** | **0.33 (0.078, 0.581)** | **0.011** |
|  | Hipp vol x MIND | 0.00001 (-0.0002, 0.0002) | 0.017 (-0.321, 0.356) | 0.919 |
| **Executive Composite** | (Intercept) | 1.957 (-0.211, 4.126) | 0.002 (-0.283, 0.287) | 0.076 |
|  | Hippo volume | 0.0003 (-0.001, 0.001) | 0.114 (-0.144, 0.373) | 0.528 |
|  | MIND diet score | 0.014 (-0.058, 0.087) | 0.045 (-0.205, 0.295) | 0.697 |
|  | Age | **-0.04 (-0.066, -0.013)** | **-0.37 (-0.621, -0.12)** | **0.004** |
|  | Sex (Male) | -0.006 (-0.324, 0.312) | -0.011 (-0.602, 0.581) | 0.971 |
|  | Education | 0.05 (-0.039, 0.138) | 0.145 (-0.113, 0.404) | 0.265 |
|  | Hipp vol x MIND | -0.00003 (-0.0002, 0.0001) | -0.073 (-0.42, 0.275) | 0.677 |
|  | | | | |
|  | **Hippocampal volume x HEI 2020 score interactions** | | |  |
| **Cognition Composite** | (Intercept) | 0.364 (-1.543, 2.27) | 0.053 (-0.227, 0.332) | 0.704 |
|  | Hippo volume | 0.00004 (-0.001, 0.001) | 0.156 (-0.104, 0.417) | 0.947 |
|  | HEI 2020 score | 0.005 (-0.004, 0.015) | 0.134 (-0.105, 0.372) | 0.273 |
|  | Age | **-0.025 (-0.048, -0.002)** | **-0.266 (-0.507, -0.025)** | **0.031** |
|  | Sex (Male) | -0.093 (-0.36, 0.174) | -0.197 (-0.763, 0.369) | 0.489 |
|  | Education | **0.087 (0.012, 0.162)** | **0.29 (0.041, 0.54)** | **0.023** |
|  | Hipp vol x HEI | 1.3x10^-6^ (-2.1x10^-5^, 2.4x10^-5^) | 0.02 (-0.306, 0.346) | 0.905 |
| **Memory Composite** | (Intercept) | -1.045 (-3.785, 1.696) | 0.062 (-0.23, 0.355) | 0.448 |
|  | Hippo volume | 0.0001 (-0.002, 0.002) | 0.116 (-0.157, 0.389) | 0.924 |
|  | HEI 2020 score | 0.008 (-0.006, 0.022) | 0.141 (-0.108, 0.391) | 0.265 |
|  | Age | -0.012 (-0.045, 0.02) | -0.095 (-0.347, 0.157) | 0.453 |
|  | Sex (Male) | -0.155 (-0.539, 0.229) | -0.239 (-0.831, 0.354) | 0.423 |
|  | Education | **0.127 (0.02, 0.234)** | **0.308 (0.048, 0.569)** | **0.021** |
|  | Hipp vol x HEI | 6.2x10^-7^ (-3.1x10^-5^, 3.3x10^-5^) | 0.007 (-0.335, 0.348) | 0.969 |
| **Executive Composite** | (Intercept) | 1.772 (-0.489, 4.033) | 0.017 (-0.274, 0.308) | 0.122 |
|  | Hippo volume | 2.0x10^-6^ (-0.002, 0.002) | 0.135 (-0.137, 0.406) | 0.998 |
|  | HEI 2020 score | 0.003 (-0.009, 0.015) | 0.064 (-0.184, 0.313) | 0.616 |
|  | Age | **-0.038 (-0.065, -0.011)** | **-0.353 (-0.604, -0.102)** | **0.007** |
|  | Sex (Male) | -0.031 (-0.348, 0.286) | -0.058 (-0.647, 0.532) | 0.845 |
|  | Education | 0.047 (-0.041, 0.136) | 0.138 (-0.121, 0.398) | 0.289 |
|  | Hipp vol x HEI | 2.1x10^-6^ (-2.4x10^-5^, 2.8x10^-5^) | 0.026 (-0.313, 0.366) | 0.877 |

**Supplemental Table 6.** Interactions between hippocampal volume and diet scores predicting cognition

Multivariable linear regression models tested interaction terms between hippocampal volume and diet scores (MIND, HEI-2020) predicting cognitive composite outcomes (cognition, memory, executive function), adjusted for age, sex, and education. Values represent the unstandardized (B) and standardized (β) beta coefficients with 95% confidence intervals (CI) and p-values for each predictor in each model. Boldface indicates significant predictor (p<0.05).

Cognitive composite scores were created from multiple cognitive assessments. Hippocampal volume was adjusted for intracranial volume using the residual method.

Hippocampal volume did not significantly interact with either diet score, MIND or HEI 2020, in predicting cognition.

| Cortex volume x diet score → cognition interactions  Models: lm(cognitive composite ~ cortex volume x diet score + covariates) | | | | |
| --- | --- | --- | --- | --- |
| **Outcome** | **Predictor** | **B (95% CI)** | **β (95% CI)** | **p-value** |
|  | **Cortex volume x MIND diet score interactions** | | |  |
| **Cognition Composite** | (Intercept) | 0.111 (-1.591, 1.814) | 0.062 (-0.194, 0.317) | 0.896 |
|  | Cortex volume | **1.9x10^-5^ (4.7x10^-6^, 3.3x10^-5^)** | **0.191 (-0.043, 0.425)** | **0.010** |
|  | MIND diet score | 0.056 (-0.002, 0.113) | 0.214 (-0.012, 0.44) | 0.057 |
|  | Age | **-0.026 (-0.047, -0.004)** | **-0.272 (-0.503, -0.042)** | **0.021** |
|  | Sex (Male) | -0.091 (-0.342, 0.159) | -0.193 (-0.724, 0.337) | 0.468 |
|  | Education | **0.114 (0.048, 0.18)** | **0.38 (0.159, 0.6)** | **0.001** |
|  | Cortex vol x MIND | -2.8x10^-6^ (-5.2x10^-6^, -4.1x10^-7^) | **-0.30 (-0.556, -0.044)** | **0.023** |
| **Memory Composite** | (Intercept) | -1.303 (-3.801, 1.194) | 0.051 (-0.222, 0.323) | 0.300 |
|  | Cortex volume | 1.7x10^-5^ (-3.6x10^-6^, 3.8x10^-5^) | 0.09 (-0.159, 0.34) | 0.103 |
|  | MIND diet score | **0.098 (0.013, 0.182)** | **0.275 (0.034, 0.516)** | **0.024** |
|  | Age | -0.015 (-0.046, 0.017) | -0.115 (-0.36, 0.131) | 0.354 |
|  | Sex (Male) | -0.106 (-0.474, 0.261) | -0.164 (-0.731, 0.403) | 0.564 |
|  | Education | **0.157 (0.06, 0.254)** | **0.381 (0.145, 0.617)** | **0.002** |
|  | Cortex vol x MIND | -2.7x10^-6^ (-6.2x10^-6^, 7.9x10^-7^) | -0.212 (-0.486, 0.062) | 0.126 |
| **Executive Composite** | (Intercept) | 1.526 (-0.538, 3.591) | 0.047 (-0.225, 0.319) | 0.144 |
|  | Cortex volume | **2.1x10^-5^ (3.3x10^-6^, 3.8x10^-5^)** | **0.226 (-0.023, 0.475)** | **0.021** |
|  | MIND diet score | 0.014 (-0.056, 0.084) | 0.044 (-0.196, 0.284) | 0.688 |
|  | Age | **-0.036 (-0.063, -0.01)** | **-0.34 (-0.585, -0.095)** | **0.007** |
|  | Sex (Male) | -0.076 (-0.38, 0.228) | -0.142 (-0.707, 0.423) | 0.617 |
|  | Education | 0.071 (-0.009, 0.151) | 0.207 (-0.028, 0.442) | 0.083 |
|  | Cortex vol x MIND | -2.9x10^-6^ (-5.8x10^-6^, 1.9x10^-8^) | -0.271 (-0.544, 0.002) | 0.051 |
|  | | | | |
|  | **Cortex volume x HEI 2020 score interactions** | | | |
| **Cognition Composite** | (Intercept) | -0.027 (-1.885, 1.83) | 0.1 (-0.164, 0.365) | 0.977 |
|  | Cortex volume | 2.4x10^-5^ (1.4x10^-6^, 4.6x10^-5^) | 0.175 (-0.068, 0.418) | 0.038 |
|  | HEI 2020 score | 0.004 (-0.005, 0.014) | 0.098 (-0.132, 0.329) | 0.379 |
|  | Age | **-0.023 (-0.046, -0.001)** | **-0.25 (-0.489, -0.012)** | **0.040** |
|  | Sex (Male) | -0.122 (-0.377, 0.132) | -0.259 (-0.798, 0.279) | 0.339 |
|  | Education | **0.121 (0.05, 0.192)** | **0.403 (0.166, 0.641)** | **0.001** |
|  | Cortex vol x HEI | -3.5x10^-7^ (-7.2x10^-7^, 2.2x10^-8^) | -0.23 (-0.473, 0.014) | 0.065 |
| **Memory Composite** | (Intercept) | -1.437 (-4.138, 1.265) | 0.1 (-0.18, 0.38) | 0.291 |
|  | Cortex volume | 3.0x10^-5^ (0,-2.8x10^-6^, 6.2x10^-5^) | 0.084 (-0.173, 0.342) | 0.073 |
|  | HEI 2020 score | 0.007 (-0.007, 0.021) | 0.123 (-0.121, 0.367) | 0.304 |
|  | Age | -0.012 (-0.045, 0.02) | -0.097 (-0.349, 0.156) | 0.446 |
|  | Sex (Male) | -0.17 (-0.54, 0.2) | -0.262 (-0.832, 0.309) | 0.362 |
|  | Education | **0.169 (0.065, 0.272)** | **0.409 (0.157, 0.661)** | **0.002** |
|  | Cortex vol x HEI | -4.6x10^-7^ (-1.0x10^-6^, 7.2x10^-8^) | -0.224 (-0.482, 0.035) | 0.088 |
| **Executive Composite** | (Intercept) | 1.382 (-0.847, 3.612) | 0.055 (-0.223, 0.334) | 0.219 |
|  | Cortex volume | 1.8x10^-5^ (-9.1x10^-6^, 4.5x10^-5^) | 0.206 (-0.05, 0.462) | 0.190 |
|  | HEI 2020 score | 0.001 (-0.01, 0.013) | 0.024 (-0.219, 0.268) | 0.827 |
|  | Age | **-0.034 (-0.061, -0.008)** | **-0.323 (-0.574, -0.072)** | **0.013** |
|  | Sex (Male) | -0.075 (-0.381, 0.23) | -0.14 (-0.708, 0.428) | 0.624 |
|  | Education | 0.073 (-0.012, 0.159) | 0.215 (-0.036, 0.465) | 0.091 |
|  | Cortex vol x HEI | -2.3x10^-7^ (-6.7x10^-7^, 2.1x10^-7^) | -0.133 (-0.39, 0.124) | 0.303 |

**Supplemental Table 7.** Interactions between cortex volume and diet scores predicting cognition

Multivariable linear regression models tested interaction terms between cortical volume and diet scores (MIND, HEI-2020) predicting cognitive composite outcomes (cognition, memory, executive function), adjusted for age, sex, and education. Values represent the unstandardized (B) and standardized (β) beta coefficients with 95% confidence intervals (CI) and p-values for each predictor in each model. Boldface indicates significant predictor (p<0.05).

Cognitive composite scores were created from multiple cognitive assessments. Cortex volume was adjusted for intracranial volume using the residual method.

Cortex volume significantly interacted with the MIND diet score in predicting overall cognition but was only marginally significant for memory and executive function. Cortex volume did not significantly interact with the HEI 2020 score to predict cognition.

|  | **Composite Cognition** | | **Memory Composite** | | **Executive Composite** | |
| --- | --- | --- | --- | --- | --- | --- |
| **Diet score** | **β (95% CI)** | **p-value** | **β (95% CI)** | **p-value** | **β (95% CI)** | **p-value** |
| **White matter hyperintensity (WMH) volume** | | | | | | |
| Low MIND = 3.8 | **-0.62**  **(-0.86, -0.39)** | **<0.001** | **-0.43**  **(-0.70, -0.16)** | **0.002** | **-0.62**  **(-0.87, -0.36)** | **<0.001** |
| Mean MIND = 5.7 | -0.15  (-0.35, 0.05) | 0.14 | -0.08  (-0.32, 0.15) | 0.47 | -0.17  (-0.39, 0.05) | 0.12 |
| High MIND = 7.5 | 0.32  (-0.002, 0.64) | 0.051 | 0.26  (-0.11, 0.63) | 0.17 | 0.27  (-0.08, 0.62) | 0.13 |
|  | | | | | | |
| **Cortex volume** | | | | | | |
| Low MIND = 3.7 | **0.44**  **(0.12, 0.77)** | **0.008** | - | - | - | - |
| Mean MIND = 5.6 | 0.17  (-0.04, 0.38) | 0.11 | - | - | - | - |
| High MIND = 7.4 | -0.10  (-0.40, 0.20) | 0.52 | - | - | - | - |

**Supplemental Table 8.** Simple slopes analyses for significant pathology x diet score interactions

Simple slopes were estimated for interaction terms considered statistically significant (p<0.05) in interaction models adjusted for age, sex, and education. These analyses quantify associations between structural brain pathology measures (WMH and cortical volume) and cognitive outcomes (composite cognition, memory composite, executive composite) conditional on diet score using three specific values: (-1 SD), mean, and high (+1 SD) levels of dietary adherence. All continuous variables were z-scored to derive the standardized coefficients.

Values shown are the standardized beta estimate (β) with 95% confidence intervals (CI), representing the relationship between the pathology marker and cognition (slope) at that particular diet score level, and p-values, testing whether the partial regression line significantly differs from zero. Boldface indicates statistically significant slopes (p<0.05). Dashes (-) indicate that simple slopes were not estimated due to non-significant interaction terms.

Cognitive composite scores were created from multiple cognitive assessments. WMH was cube-root transformed. Cortex volume was adjusted for intracranial volume using the residual method.
